# Macro–Meso-Scale Simulation for Surface Roughness Evolution of Aluminum Alloy Tube Drawing Process

**DOI:** 10.3390/ma19173568

**Published:** 2026-08-22

**Authors:** Chengshang Liu, Yijing Shao, Yang Song, Wenxin Yu, Wujiao Xu

**Affiliations:** 1 College of Material Science and Engineering, Chongqing University, Chongqing 400044, China; caser_liu@163.com (C.L.); ssshaoyijing@163.com (Y.S.); shuritto_song@163.com (Y.S.); 2 Chongqing Chuanyi Automation Co., Ltd., Chongqing 401121, China

**Keywords:** macro–meso-scale simulation, surface roughness, crystal plasticity finite element modelling, fluid–solid interaction modelling, aluminum alloy tube drawing

## Abstract

Surface roughening is a common defect in plastic deformation processing, directly affecting product surface quality and service performance. This study investigates the mechanisms of surface roughness evolution during plastic deformation by considering both intrinsic and extrinsic factors. A macro–meso-scale modelling framework is developed by coupling crystal plasticity finite element modelling, fluid–solid interaction modelling, and macro–meso boundary conditions. The crystal plasticity model incorporates a constitutive model based on crystal plasticity theory, a Voronoi-based geometric model, and a real rough-surface topography model to capture non-uniform grain-scale plastic deformation. Fluid–solid interaction modelling is introduced to analyze the influence of liquid lubricant on the deforming solid material. Boundary interpolation and continuous displacement theories are then used to transfer macro-scale boundary constraints to the meso scale. The proposed framework is numerically implemented and applied to the aluminum alloy tube drawing process. The effects of intrinsic factors, including grain size, grain orientation, and initial surface roughness, and extrinsic factors, including deformation path, strain rate, and lubrication condition, are systematically examined. From a practical point of view, effective strategies to improve surface quality are by reducing grain size, lowering initial surface roughness, decreasing the strain rate and using low-viscosity lubricants.

## 1. Introduction

For some critical components in special engineering applications, such as waveguide tube of radar [1], photoconductive tube of printer cartridge [2], stainless steel tube of semiconductor production equipment [3] and rolled copper foil of printed circuit board [4], etc., their working surfaces are often required to have extremely high surface quality due to the specific conditions of use. Due to the limitations of the product geometry, it is difficult to further treat the surface quality by physical grinding or chemical polishing after plastic forming, so plastic forming is the most suitable manufacturing method [5,6,7]. Therefore, a fundamental understanding of the mechanisms underlying surface roughness evolution under plastic deformation is essential.

According to ISO 25178-2 [8], surface texture is composed of three components: roughness, waviness, and form. The present study focuses on roughness—the short-wavelength component—which is characterized by the root mean square height parameter *Sq*. The determinants of surface roughness in plastic forming processes can be classified into two categories: internal and external factors. Internal factors originate from the material’s own characteristics during deformation, such as grain size, grain orientation, and the initial roughness of the workpiece. External factors involve influences imposed from outside the material, including deformation path, accumulated strain, and lubrication condition.

Previous studies have demonstrated that material-related parameters—such as grain size, grain orientation, and initial surface roughness—are strongly correlated with the final surface roughness after deformation. Wouters et al. [9] and Leu [10] examined the influence of grain size on surface roughness under uniaxial tension and established a linear relationship between these two variables. Mahmudi et al. [11] analyzed the roughening behaviour of copper alloys during tensile loading and observed that the rate of surface roughness change increased with larger grain sizes, a finding consistent with the classical empirical formula. Zamiri et al. [12] explored the role of grain orientation in surface roughness development; their simulation results indicated that polycrystals with a weak surface texture exhibited pronounced roughening after plastic deformation. Wouters et al. [13] further investigated the connection between the orientation factor and surface roughness, revealing that roughness was governed not only by the orientation of surface grains but also by that of subsurface grains. Zhang et al. [14] studied the roughness evolution of aluminum alloy with varying initial roughness under uniaxial tension. They decomposed the total roughness change into two components: surface flattening arising from homogeneous deformation and surface roughening caused by inhomogeneous deformation. Their findings showed that a lower initial surface roughness led to a greater contribution from inhomogeneous deformation, thereby resulting in a higher rate of roughness change.

Meanwhile, extensive research has been conducted on the effects of external parameters—such as accumulated strain, strain rate, and lubrication conditions—on post-deformation surface roughness. Dai and Chiang [15] performed experimental investigations into how deformation paths influence the surface roughness of copper alloys, and their results indicated that the deformation path plays a critical role in roughness evolution. Becker [16] developed a crystal plasticity-based finite element model to predict surface roughness changes under various deformation paths, and the simulations confirmed that the deformation path significantly alters the resulting roughness. Lo and Horng [17] observed that for AA1050-O plates subjected to plastic deformation, surface roughness varied linearly with strain but remained unaffected by strain rate. In contrast, Wu and Lloyd [18] reported that an increase in strain rate led to a slight reduction in surface roughness. Meanwhile, Shi [19] noted that surface roughness increased with higher loading rates. Li et al. [20] examined the role of lubrication conditions during uniaxial plane compression and discovered that under dry friction, the surface topography becomes progressively flatter as plastic deformation increases, whereas the application of a lubricant substantially suppresses this flattening effect. Kucharski et al. [21] conducted compression tests using artificially introduced single pits and found that the deformation behaviour of these pits differed markedly between lubricated and dry friction conditions.

**Figure 1:**
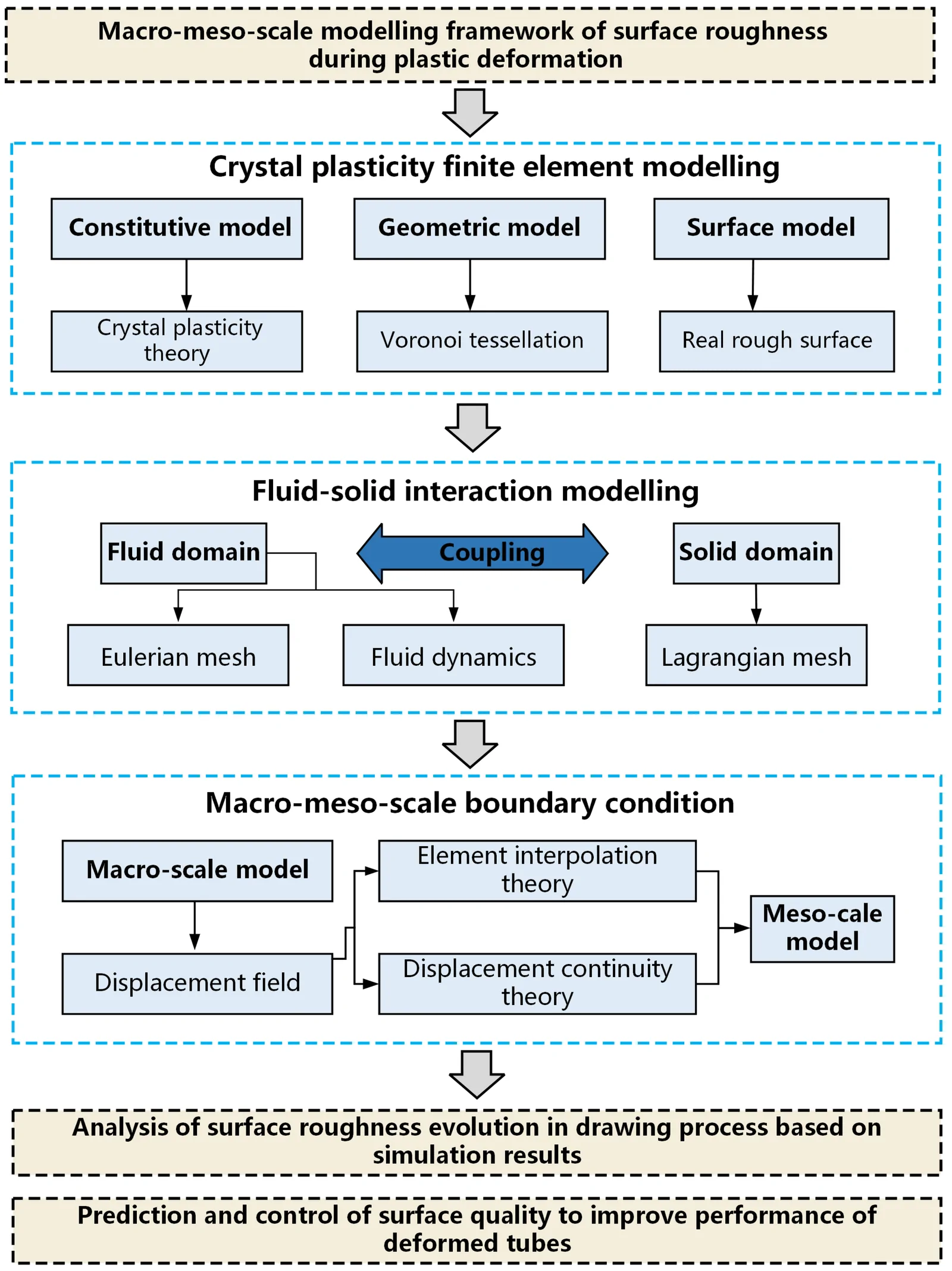
Framework of macro–meso-scale modelling.

To achieve a comprehensive understanding of the mechanisms governing surface roughness evolution under the combined influence of material-related and process-related parameters, this paper develops a multi-scale modelling framework that spans both macroscopic and mesoscopic scales. As illustrated in Figure 1, the proposed framework integrates three key components: a crystal plasticity finite element model, a fluid–solid interaction model, and a macro-to-meso scale boundary condition. Specifically, the crystal plasticity finite element model is first constructed, incorporating a constitutive law derived from crystal plasticity theory, a geometric representation based on Voronoi tessellation, and a surface topography model built upon realistic rough surfaces. This component aims to capture grain-scale inhomogeneous plastic deformation. Subsequently, a fluid–solid interaction model is employed to examine how liquid-state lubricants affect the deformation behaviour of solid-state materials. Next, boundary interpolation theory and continuous displacement theory are utilized to transfer macroscopic boundary constraints down to the mesoscopic level, thereby establishing the macro–meso boundary condition. Finally, the entire framework is numerically implemented and applied to the tube drawing process of aluminum alloys. Using this approach, the influences of both intrinsic factors (including grain size, grain orientation, and initial surface roughness) and extrinsic factors (such as deformation path, strain rate, and lubrication condition) on surface roughness evolution are systematically investigated.

## 2. Crystal Plasticity Finite Element Modelling

Crystal plasticity finite element modelling (CPFEM), which combines crystal plasticity theory with finite element theory, is a cutting-edge method for analyzing the plastic deformation behaviour of single crystal and polycrystals at the meso-scale. CPFEM has been widely used in the popular fields of plastic deformation analysis at the meso-scale and advanced plastic processing, which is one of the most suitable tools to study the texture evolution, local inhomogeneous deformation and surface roughness evolution of metallic materials. In this chapter, a CPFEM is built which couples the crystal plasticity constitutive model, the geometric model of the polycrystal and the rough surface model.

### 2.1. Crystal Plasticity Constitutive Model

The crystal plasticity constitutive model originally proposed by Taylor [22,23], Polanyi [24], Orowan [25], Schmid [26], Hill [27], Rice [28], and Asaro [29] was adopted in the present study. The total deformation gradient F of crystalline materials is decomposed into two distinct physical mechanisms: the elastic part $F^{e}$ and the plastic part $F^{p}$. The elastic deformation is attributed to lattice distortion and rigid-body rotation of the crystal, whereas the plastic deformation is represented by a series of shear deformation events occurring along specific crystallographic slip systems—comprising particular planes and directions—driven by dislocation glide (or deformation twinning).

(1) $$F=F^{e}F^{p}$$

**Figure 2:**
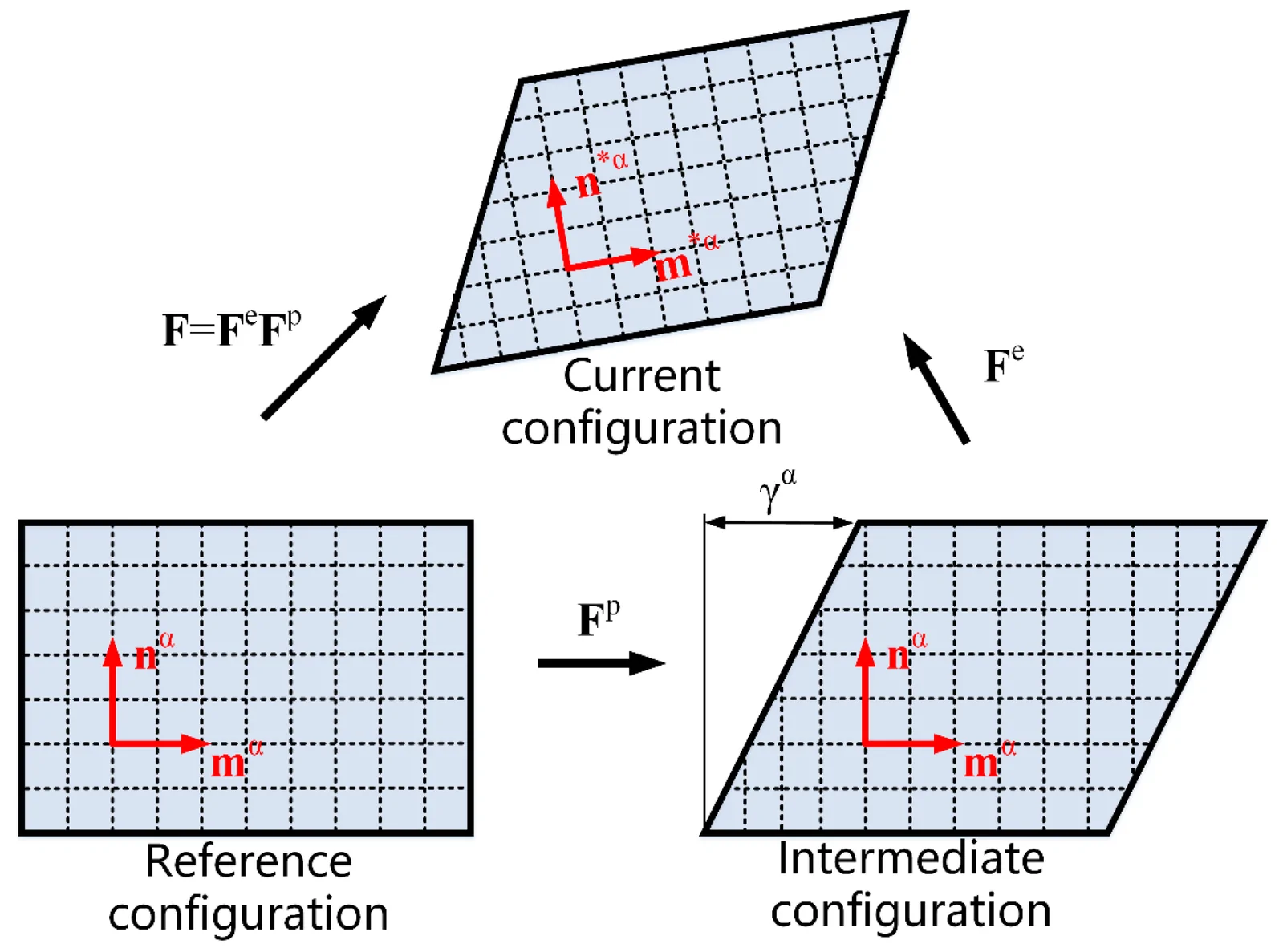
Schematic representation of the three configurations and the corresponding decomposition of deformation gradient into the elastic and plastic parts.

The above equation represents the total deformation (F) of the crystal divided into two steps: the reference configuration leads to an intermediate configuration through a plastic deformation gradient ($F^{p}$), which then changes to the current configuration through the elastic deformation gradient ($F^{e}$). As shown in Figure 2, the lattice vector remains unchanged at the first step, while at the second step, the lattice vector is elongated and rotated.

During the first step, the crystal only undergoes dislocation slip along the slip system, the slip direction ($m^{\alpha }$) and slip plane normal direction ($n^{\alpha }$) do not change. Therefore, the unit vectors $m^{\alpha }$ and $n^{\alpha }$ remain unchanged, which always satisfies the orthogonality relation:

(2) $$m^{\alpha }\cdot n^{\alpha }=0$$

The plastic deformation gradient ($F^{p}$) can be expressed as:

(3) $$F^{p}=\sum_{\alpha =1}^{N}{F^{p}}^{\left(\alpha \right)}=\sum_{\alpha =1}^{N}\left(I+\gamma^{\alpha }m^{\alpha }⨂n^{\alpha }\right)$$

where N denotes the sum of all slip systems of the crystal during the deformation process. For example, for a face-centred-cubic (FCC) lattice crystal, the crystal has 12 slip systems, i.e., N=12. ${F^{p}}^{\left(\alpha \right)}$ denotes the contribution of the slip system α to the overall deformation gradient $F^{p}$. I denotes the unit tensor of second order; $\gamma^{\alpha }$ is the shear strain occurring on the slip system α, ⨂ denotes the Kronecker tensor product.

During the second step, the lattice distortion and the rotation of the crystal occur due to the elastic deformation ($F^{e}$), causing the slip direction unit vector $m^{\alpha }$ and the slip plane normal unit vector $n^{\alpha }$ undergoes distortional and rotational transformations, which satisfy:

(4) $$\left\{\begin{array}{l} m^{*\alpha }=F^{e}m^{\alpha }\\ n^{*\alpha }=n^{\alpha }{F^{e}}^{-1} \end{array}\right.$$

where $m^{*\alpha }$ and $n^{*\alpha }$ denote the vector of the slip direction and slip plane normal direction at the second step, as shown in Figure 1. It can be found that when the crystal is subjected to the elastic deformation gradient $F^{e}$, $m^{*\alpha }$ and $n^{*\alpha }$ become non-unit vectors, but they still satisfy the orthogonal geometric relations.

The velocity gradient tensor L is defined as the derivative of the deformation velocity with respect to position, it can also be obtained from the deformation gradient as follows:

(5) $$L=\dot{F}F^{-1}={\dot{F}}^{e}{F^{e}}^{-1}+F^{e}\left({\dot{F}}^{p}{F^{p}}^{-1}\right){F^{e}}^{-1}$$

where the mathematical symbol (˙) and ${\left(\right)}^{-1}$ indicate the time derivative and the inverse, respectively. The plastic part of velocity gradient ($L^{p}$) is given by:

(6) $${L^{p}=\dot{F}}^{p}{F^{p}}^{-1}=\sum_{\alpha =1}^{N}{\dot{\gamma }}^{\alpha }\left(m^{\alpha }⨂n^{\alpha }\right)$$

The connection between the deformation gradient, velocity gradient, the slip direction and the slip plane normal direction of the crystal is established by (1)–(6), which are the basic kinematic equations× for the crystal deformation.

Within the framework of crystal plasticity theory applied to the intermediate configuration illustrated in Figure 1, the existence of an elastic potential was hypothesized by Hill and Rice [30]. This assumption implies that the elastic properties of the crystal remain unaffected by slip-induced shear deformation. Accordingly, the elastic constitutive relation for the crystal can be formulated as follows:

(7) $${\hat{\sigma }}^{e}=C:D^{e}$$

In this expression, ${\hat{\sigma }}^{e}$ denotes the corotational stress rate of the intermediate configuration (the Jaumann rate of Kirchhoff stress), and $D^{e}$ indicates the elastic part of the deformation rate tensor D. C($C_{ijkl}=C_{jikl}=C_{ijlk}=C_{klij}$) is the elastic modulus tensor, and for cubic crystal structures C is determined by the three independent variables $C_{11}$, $C_{12}$ and $C_{44}$.

The slip shear rate (${\dot{\gamma }}^{\alpha }$) is a prerequisite for solving for the stress rate and deformation rate, which need to be calculated using a hardening constitutive model. In the context of the rate-dependent constitutive formulation, a power-law relationship was proposed by Hutchinson [31] to describe the slip shear rate ${\dot{\gamma }}^{\alpha }$:

(8) $${\dot{\gamma }}^{\alpha }={\dot{\gamma }}_{0}{\left|\frac{\tau^{\alpha }}{g^{\alpha }}\right|}^{\frac{1}{m}}sgn\left(\tau^{\alpha }\right)$$

Here, ${\dot{\gamma }}_{0}$ denotes a reference strain rate, assumed identical across all twelve slip systems characteristic of an FCC lattice. The parameter m represents the strain-rate sensitivity, while corresponds to the resolved shear stress acting on slip system α. The function $g^{\alpha }$ is a strain-hardening term that quantifies the resistance to dislocation motion during plastic deformation. Its evolution is governed by a hardening law [32], expressed as:

(9) $${\dot{g}}^{\alpha }=\sum_{\beta }h_{\alpha \beta }\left|{\dot{\gamma }}^{\beta }\right|$$

where $h_{\alpha \beta }$ is the hardening matrix describing self- and latent-hardening interactions among slip systems.

(10) $$h_{\alpha \beta }=[1+(1-q)\delta_{\alpha \beta }]h_{\alpha \alpha }$$

In the above expression, q denotes an interaction coefficient characterizing the latent hardening behaviour of the crystal, while $\delta_{\alpha \beta }$ represents the Kronecker delta. The self-hardening function $h_{\alpha \alpha }$ depends on the total accumulated shear strain γ within the slip system [33], and is given by:

(11) $$h_{\alpha \alpha }=h_{0}{sech}^{2}\left|\frac{h_{0}\gamma }{\tau_{s}-\tau_{0}}\right|$$

where $h_{0}$, $\tau_{0}$, and $\tau_{s}$ are material hardening constants. Specifically, $h_{0}$ is the initial hardening rate, $\tau_{0}$ is the yield stress corresponding to the initial value of the current strength $g^{\alpha }(0)$, and $\tau_{s}$ is the saturation shear stress. The notation sech refers to the hyperbolic secant function.

The elastic parameters for the crystal plasticity constitutive model of 6061 aluminum alloy were adopted from the literature [34]. The plastic parameters employed in this study are listed in Table 1 and were obtained through calibration against the stress–strain curve. Specifically, the plastic parameters ($h_{0}$, $\tau_{0}$, $\tau_{s}$, m, q) were obtained by fitting the simulated uniaxial tensile stress–strain curve to experimental data from standard tensile tests, following the methodology in the literature [35,36]. The initial hardening rate $h_{0}$ was adjusted to match the plastic slope, while $\tau_{0}$ and $\tau_{s}$ were determined from the initial yield stress and saturation stress, respectively. The strain rate sensitivity exponent m=0.05 and the interaction constant q=1.1 were typical value for AA6061 [14,37]. The complete set of crystal plasticity constitutive equations was implemented in the commercial finite-element software Abaqus 6.14/Standard 13.0 by means of a user-defined material subroutine.

### 2.2. Geometric Model and Meshing Method

To analyze the deformation behaviour of polycrystalline materials, a geometric representation of the grain structure must be constructed. In the present work, the Voronoi tessellation technique [38] was employed to create the polycrystalline microstructure, and the polycrystalline geometries constructed by the Voronoi tessellation method can capture information such as geometric details and size distribution of the real grains [39]. The construction process of Voronoi tessellation is similar to the crystallization and growth process of grains in metallic materials. And the Voronoi tessellation method can control the information on grain shape, grain size and grain distribution according to the researcher’s needs, so this method is widely used to model the geometry of polycrystalline materials [40,41,42,43,44,45,46,47,48,49,50,51].

The fundamental concept of the Voronoi tessellation method involves partitioning a spatial domain according to the principle of nearest distance. Taking the construction of a three-dimensional Voronoi tessellation as an illustration, let $S=\{p_{1},p_{2},\ldots p_{m}\}$ represent a set of m distinct points in three-dimensional space, referred to as seed points. Voronoi tessellation is then defined as follows:

(12) $$V\left(p_{i}\right)=\underset{i\neq j}{⋂}\left\{p\left|d(p,p_{i})<d(p,p_{j})\right.\right\},\left(i\neq j,i,j=1,2\ldots m\right)$$

where $V(p_{i})$ is the Voronoi tessellation of seed point $p_{i}$, $d(p,p_{i})$ denotes the Euclidean distance from point p to $p_{i}$. Each position within the Voronoi tessellation $V(p_{i})$ has a distance to seed point $p_{i}$ that is less than the distance to any other seed point $p_{j}$, which is also known as the closest distance principle. Each point $p_{i}$ in the set $S=\{p_{1},p_{2},\ldots p_{m}\}$ generates a corresponding Voronoi tessellation representing a grain in the polycrystalline structure.

**Figure 3:**
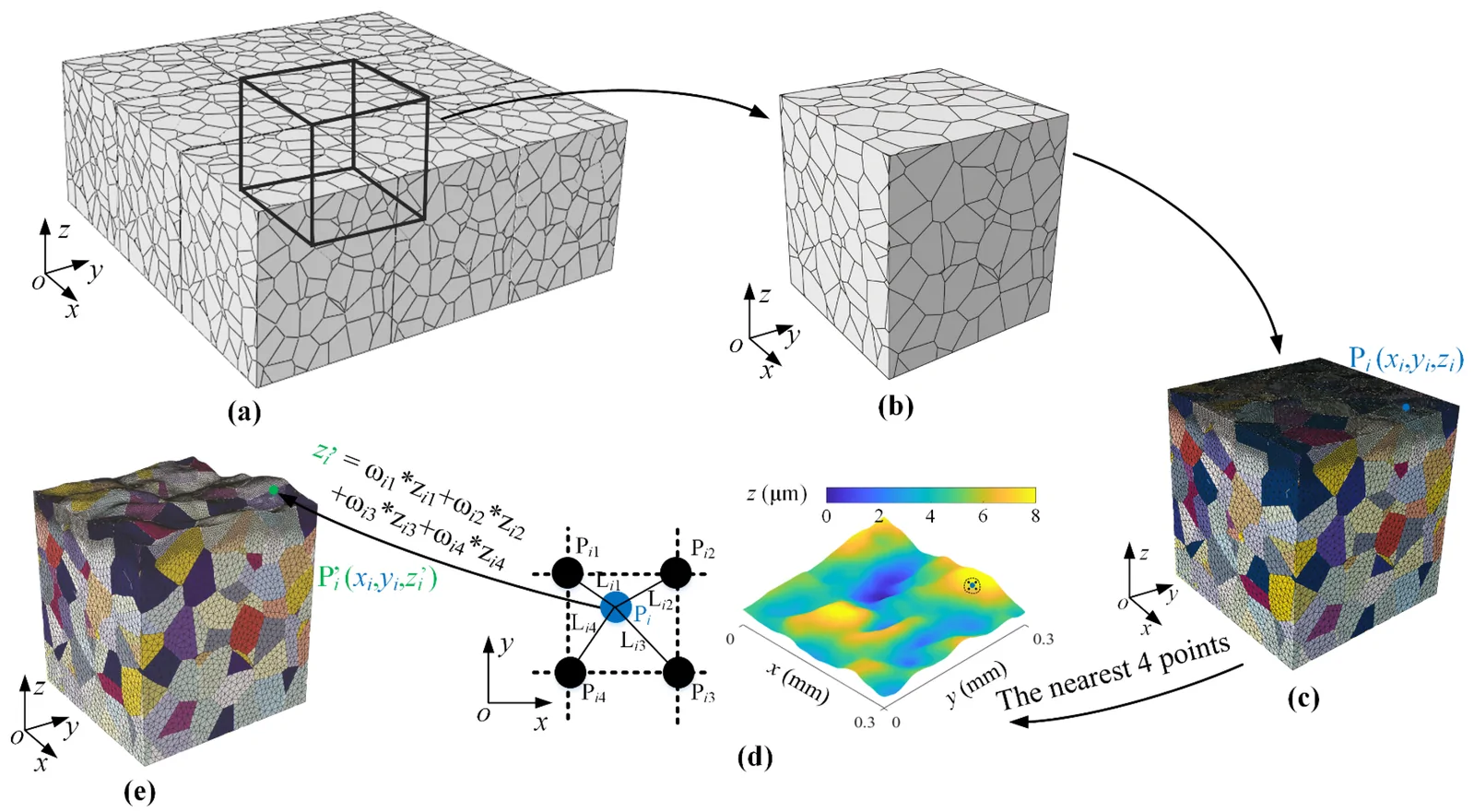
Construction process for CPFEM: (**a**) Voronoi tessellation, (**b**) periodic geometric model, (**c**) meshing method, (**d**) mapping relationship, (**e**) surface topography model.

The 3D periodic geometry model based on Voronoi tessellation in this paper is shown in Figure 3. First, a number of seed points are randomly generated in 3D space. Then, these seed points are replicated periodically in the x and y directions, and the Voronoi tessellation is generated based on the replicated periodic seed points, as shown in Figure 3a. Finally, as shown in Figure 3b, the middlemost cell is cut out as the geometric model to ensure that the obtained Voronoi tessellation has a periodic character.

The grain geometric model constructed based on Voronoi tessellation is shown in Figure 3b, which represents a cube with an edge length of 0.3 mm of the workpiece. To achieve an optimal trade-off between computational cost and numerical accuracy, a localized mesh refinement strategy was employed. Three mesh densities were tested: coarse (347,946 elements), medium (783,964 elements), and fine (1,821,838 elements). The difference in surface roughness between the coarse and medium meshes was approximately 10%, while the difference between the medium and fine meshes was less than 2%. Although the fine mesh provided slightly higher accuracy, its computational time was approximately four times that of the medium mesh. Since the computational time for the medium mesh remained acceptable while achieving a good balance between accuracy and efficiency (less than 2% error compared to the fine mesh), the medium mesh (783,964 elements) was adopted for all subsequent simulations. The adopted mesh comprises 783,964 four-node tetrahedral elements, as illustrated in Figure 3c; notably, the element size on the surface is approximately one-fifth of that at the bottom.

### 2.3. Surface Topography Model

Workpieces are often subject to a combination of many factors during production process, so that the surface of the workpiece retains the geometry of the machining marks. As a result, ideal planes do not exist in practice, and actual workpiece surfaces are rough surfaces. In order to accurately analyze the influence of various factors on the surface topography during plastic deformation, it is necessary to give the constructed model a rough surface topography.

In this paper, a method of constructing a surface topography using the real rough surface of the workpiece as a finite element model is presented. The construction procedure comprises three sequential stages, as illustrated in Figure 3c–e. Initially, a meshed model featuring a perfectly planar surface is generated (Figure 3c), containing the positional coordinates of all nodal points. Subsequently, topographic measurement data representing the actual surface profile are prepared in the form of a 1024 × 1024 matrix (Figure 3d). For each node $P_{i}\left(x_{i},y_{i},z_{i}\right)$ in the flat-surface model, the four nearest measurement points in the *xoy*-plane—denoted as $P_{i1}$, $P_{i2}$, $P_{i3}$ and $P_{i4}$—are identified based on Euclidean distance (Figure 3d). Finally, a mapping relationship is established between the measured heights and the model coordinates: the updated height ${z_{i}}^{\prime }$ of node $P_{i}$ at location $\left(x_{i},y_{i}\right)$ is computed from the measured elevation data, subject to the following condition:

(13) $${z_{i}}^{\prime }=\sum_{j=1}^{j=4}\omega_{ij}\cdot z_{ij}$$

In this expression, ${z_{i}}^{\prime }$ denotes the measured height at the sampling point $P_{ij}$ (where *j* = 1, 2, 3, 4), and $\omega_{ij}$ represents the weighting coefficient determined by the distance between node $P_{i}$ and the sampling point $P_{ij}$, expressed as:

(14) $$\left\{\begin{array}{l} \omega_{i1}=1-\frac{L_{i1}}{L_{i}}\\ \omega_{i2}=\frac{L_{i1}}{L_{i}}\left(1-\frac{L_{i2}}{L_{i}}\right)\\ \omega_{i3}=\frac{L_{i1}}{L_{i}}\frac{L_{i2}}{L_{i}}\left(1-\frac{L_{i3}}{L_{i}}\right)\\ \omega_{i4}=\frac{L_{i1}}{L_{i}}\frac{L_{i2}}{L_{i}}\frac{L_{i3}}{L_{i}} \end{array}\right.$$

Here, $L_{ij}$ is the Euclidean distance projected onto the *xoy*-plane between node $P_{i}$ and sampling point $P_{ij}$, while $L_{i}$ is the cumulative sum of the four distances, i.e., $L_{i}=\sum_{j=1}^{j=4}L_{ij}$.

Equation (14) defines the transformation from the measured surface data to the model coordinate system. By applying this transformation, each node originally located at $P_{i}\left(x_{i},y_{i},z_{i}\right)$ on the perfectly flat surface (Figure 3c) is relocated to its updated position ${P_{i}}^{\prime }\left(x_{i},y_{i},{z_{i}}^{\prime }\right)$ (Figure 3e). Repeating this procedure for all nodes yields the reconstructed realistic surface model, as depicted in Figure 3e. It is evident that the detailed features of the actual surface topography—including the count and precise positions of peaks and valleys—are faithfully reproduced in the constructed model.

A CPFEM is constructed in Abaqus software by the above operations, which is suitable for simulating plastic deformation of polycrystalline materials. However, the effect of lubricant on plastic deformation is neglected in this CPFEM and it is necessary to take it into account in the model.

## 3. Fluid–Solid Interaction Modelling

Lubricant is one of the most important factors in the field of plastic deformation that cannot be ignored. Its main role is to reduce friction and wear at the contact interface between the workpiece and the die, and to extend the working life of the die. Depending on the material state, lubricants can be divided into single-phase lubricants such as gaseous, liquid and solid, as well as gas–liquid, liquid–liquid, liquid–solid two-phase lubricants and even gas–liquid–solid three-phase lubricants [52]. Of these, liquid state and solid state are the two most widely used types of lubricants in the plastic forming industry. Compared to solid state lubricant, the use of liquid state lubricant can have beneficial effects such as cooling the friction subsystem, carrying away wear debris and particulate impurities from the contact interface. Therefore, the cold drawing process for waveguide tubes studied in this paper uses a liquid lubricating medium as the lubricant at the contact interface between the workpiece and the die. The liquid lubricating medium is in the state of a fluid, while the solid die and workpiece are in the state of a solid. Therefore, the study of roughness evolution under lubricant conditions is a typical fluid–solid interaction (FSI) problem.

### 3.1. Coupled Eulerian–Lagrangian Method

**Figure 4:**
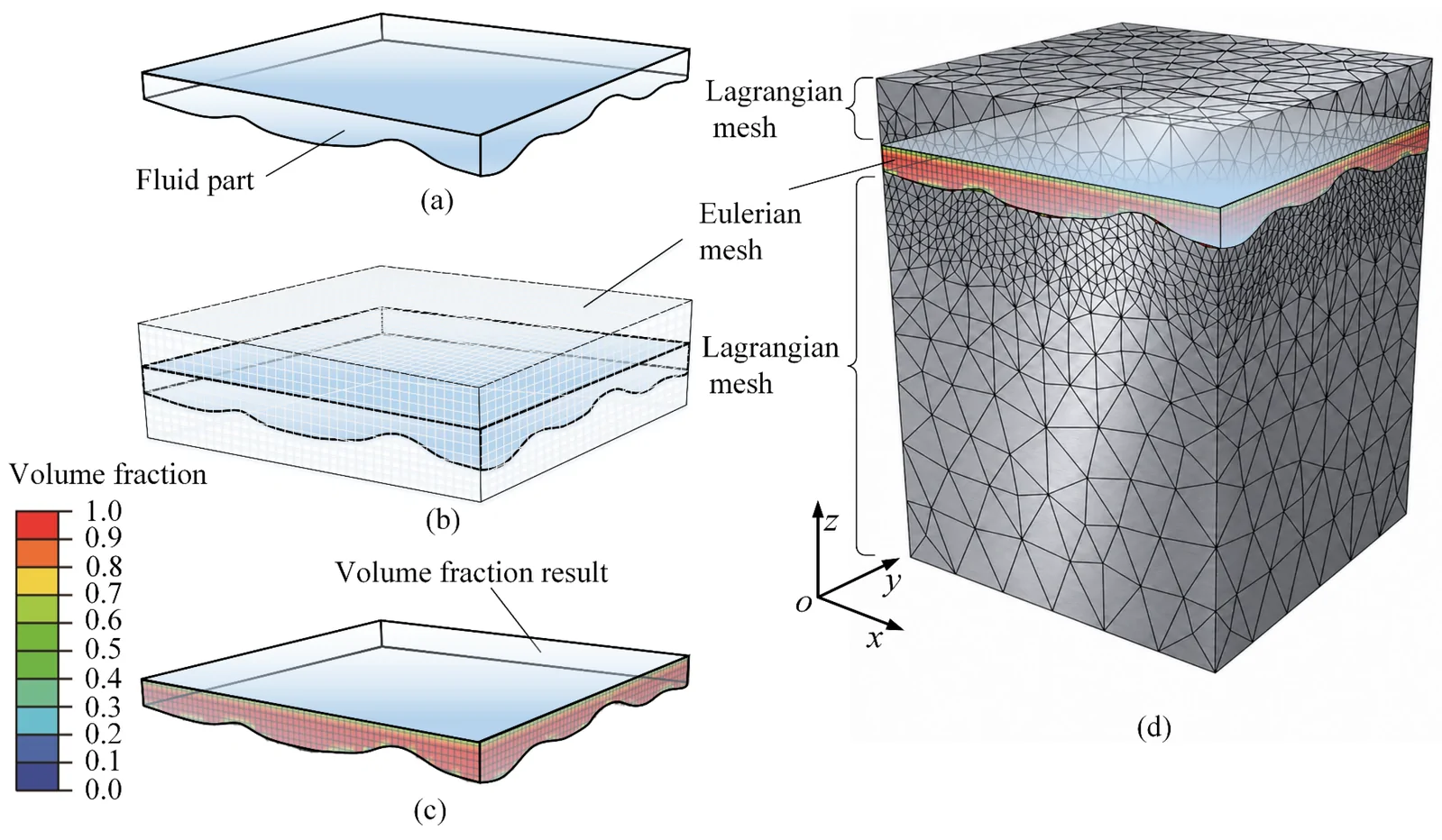
Fluid–structure interaction model constructed using the CEL approach: (**a**) spatial domain initially occupied by the fluid phase; (**b**) overlapping region between the fluid domain and the Eulerian mesh; (**c**) computation of the volume fraction; (**d**) finite element configuration wherein solid bodies are discretized with Lagrangian mesh and the fluid phase is represented by Eulerian mesh.

As the material state of die and workpiece are solid and have relatively small deformations, which are suitable for modelling with Lagrangian meshes [53]. However, the material state of the lubricant is fluid, which has large flow displacements, so Lagrangian meshes are not suitable for the modelling of the lubricant. In order to maintain the fluid model with a good accuracy in the presence of large deformations, i.e., to avoid violent mesh deformations of the fluid model, the mesh of the fluid model can be re-meshed using a mesh adaptive (Arbitrary Lagrangian Eulerian, ALE) method [54]. Nevertheless, the ALE approach becomes unsuitable when the lubricant film thickness progressively diminishes and contact conditions evolve during plastic deformation—for instance, at the initiation of solid-to-solid contact [55]. To address the fluid discontinuity issue arising from the thinning oil film, the Coupled Euler–Lagrange (CEL) method was adopted in the present study. In this scheme, the lubricating medium is discretized using a Eulerian mesh, as illustrated in Figure 4d. The contact criterion in the CEL method relies on computing the volume of fluid (VOF) [56], which reconstructs the interface between the fluid (contained within the Eulerian mesh) and the solid (represented by the Lagrangian mesh) based on the volume fraction [57]. Taking the fluid domain shown in Figure 4a as an example, the volume fraction calculation proceeds as follows. First, the fluid region is superimposed onto the discrete Eulerian mesh, resulting in partial overlap between the fluid geometry and the Eulerian elements (Figure 4b). Next, the volume fraction tool determines the proportion of each Eulerian element occupied by the fluid (Figure 4c). An element fully filled with fluid receives a volume fraction of 1, whereas an element devoid of lubricant is assigned a value of 0. At every incremental step of the CEL analysis, the volume fraction for each element is recalculated, thereby updating the evolving lubricant boundary. By integrating two distinct mesh types within a single finite element model, the CEL method is particularly effective for simulating coupled solid–solid and fluid–solid interactions, such as the constrained surface deformation under lubricated conditions examined in this work.

### 3.2. Fluid Dynamics and Material Properties

The motion of the liquid lubricant at the interface between the die and the workpiece follows the three physical conservation laws of conservation of mass, conservation of momentum and conservation of energy, and their corresponding governing equations are the basic fluid dynamics equations [55].

The conservation of mass equation is expressed in fluid dynamics as a continuity equation, which assumes that the fluid satisfies a continuous medium model where the mass of the inflowing fluid micro-element per unit time is equal to the increase in mass of that fluid micro-element, and conversely the mass of the outflowing fluid micro-element per unit time is equal to the decrease in mass of that fluid micro-element, indicating that the mass in the system does not arise out of thin air. The expression for the conservation of mass equation is as follows:

(15) $$\frac{\partial \rho }{\partial t}+\nabla (\rho V)=0$$

where ρ is the density of the fluid, and t represents time, the mathematical symbol ∇ represents the divergence differential operator; V represents the velocity vector of the fluid micro-element.

In fluid dynamics, the momentum conservation equation is embodied by the Navier–Stokes equation, which is fundamentally rooted in Newton’s second law. This principle dictates that the temporal rate of change in momentum for a fluid element is equivalent to the resultant of all external forces exerted upon it. The corresponding momentum conservation relation is given as follows:

(16) $$\rho \left(\frac{\partial V}{\partial t}+V\cdot \nabla V\right)=-\nabla p+\mu \nabla^{2}V+f$$

where p represents the hydrostatic pressure of the fluid; μ represents the dynamic viscosity of the fluid; f represents the external force vector on the fluid micro-element.

The principle of energy conservation constitutes a fundamental physical law that must be satisfied in systems involving heat transfer. It originates from the first law of thermodynamics, which asserts that the rate of energy increase within a fluid element equals the sum of the net heat influx into that element and the work performed upon it by body forces and surface tractions. The corresponding energy conservation equation is expressed as follows:

(17) $$\rho \left(\frac{\partial T}{\partial t}+\nabla \left(VT\right)\right)=\frac{k}{c_{p}}\nabla^{2}T+S_{T}$$

where T represents the temperature of the fluid; k represents the heat transfer coefficient of the fluid; $c_{p}$ represents the specific heat capacity of the fluid; and $S_{T}$ represents the viscous dissipation term of the fluid. Although the energy conservation equation is the basic controlling equation for fluid flow and heat transfer, the energy conservation equation can be disregarded for incompressible fluids where the amount of heat exchange is small and the effect of temperature changes on lubrication properties can be ignored.

The pressure and energy properties of fluid materials are usually described using the Mie–Grüneisen equation [58]:

(18) $$p-p_{ref}=\rho \Gamma (e-e_{ref})$$

In the equation, Γ is the Mie–Grüneisen ratio constant; e is the internal energy; $p_{ref}$ and $e_{ref}$ are the pressure and internal energy of the fluid in the reference state, usually at an assumed temperature of 0 K; in the reference state, $p_{ref}$ and $e_{ref}$ are independent of temperature and their values can be calculated from the Hugoniot linear relationship between the shock velocity $U_{s}$ and the particle velocity $U_{p}$ [59] as follows:

(19) $$U_{s}=c_{ref}+sU_{p}$$

where $c_{ref}$ is the speed of sound of the fluid in the reference state and s is the Hugoniot slope coefficient of the fluid. Based on the assumption of incompressible fluids, the above parameters Γ and s can be simplified to zero [53,55].

The calculation of the fluid–solid interaction problem is divided into two aspects: solid mechanics and fluid dynamics. The solid part is calculated through Equations (1)–(14) of the crystal plasticity finite element model, and the fluid part is calculated through Equations (15)–(19) of fluid dynamics. Next, an inheritance tool for cross-scale boundary condition is developed in this paper, which defines the boundary condition of a meso-scale model from the output field calculated by the macroscopic finite element model.

## 4. Macro–Meso-Scale Boundary Condition

In this study, the macroscopic scale is defined as the component level (millimetre to centimetre range), while the mesoscopic scale corresponds to the grain level (micrometre range, with an RVE edge length of 0.3 mm and grain sizes of several tens of micrometres). The basic idea of coupled macro–meso finite element simulation is to build two models at the macroscopic scale and at the mesoscopic scale. At the macroscopic scale, the object of study is meshed and the continuum with infinite degrees of freedom is transformed into a finite collection of elements, each containing several dimensions of mechanical characteristics and field information. In order for the mesoscopic model to fully reflect the properties of the macroscopic model, it is necessary to ensure that the shape and dimensions of the mesoscopic model are identical to those of the area of focus in the macroscopic model. The boundary interpolation theory and continuous displacement theory are then used to transfer the boundary constraints from the macroscopic scale to the mesoscopic scale. In this way, the mesoscopic model can completely represent the deformation state of the element of focus in the macro-scale model, thus achieving the purpose of cross-scale modelling.

The principle underlying the cross-scale finite element analysis method involves deriving meso-scale boundary conditions through the construction of a cross-scale basis function, which enables downscaled computations based on solutions obtained from the macroscopic model [60]. This technique allows for more refined analytical outcomes in critical regions when evaluating the macroscopic model response. In the present study, a cross-scale boundary condition based on nodal displacement information transfer was employed. The underlying concept is that the nodal displacement responses computed from the macroscopic model are imposed as boundary conditions on the meso-scale model, from which high-precision response results are subsequently obtained by solving the meso-scale problem.

**Figure 5:**
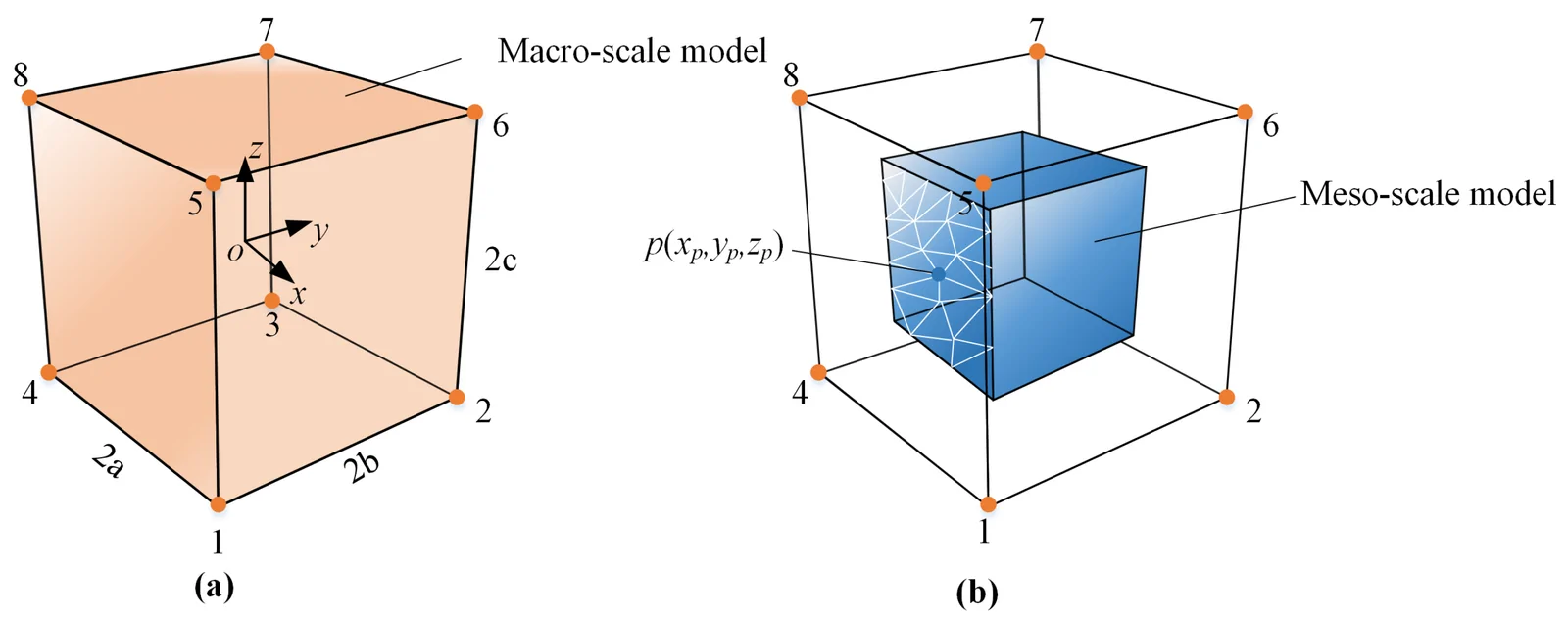
Interpolation based on finite element shape function: (**a**) eight-node hexahedron element, (**b**) obtaining the boundary conditions of the meso-scale model.

As illustrated in Figure 5, because the number of boundary nodes in the meso-scale model far exceeds that in the macro-scale model, a direct transfer of displacement information between the two scales may result in mismatched displacement data for certain boundary nodes of the meso-scale model, leading to inaccurate predictions. To resolve this issue, the finite element shape function [61,62] was adopted as the cross-scale basis function to interpolate the displacement response from the macroscopic model nodes corresponding to the boundary nodes of the mesoscopic model.

An eight-node hexahedral element is taken as an example, as shown in Figure 5a, whose geometric centre is defined as the coordinate origin, and whose sides are aligned with the x, y and z axes, with lengths of 2a, 2b and 2c respectively. If the displacements of the eight nodes along the x, y and z directions obtained from the macroscopic model are $u_{i}$, $v_{i}$ and $w_{i}$, then the displacements $u_{p}$, $v_{p}$ and $w_{p}$ along the x, y and z directions of any point $p\left(x_{p},y_{p},z_{p}\right)$ inside the hexahedral element can be expressed as:

(20) $$\left\{\begin{array}{c} u_{p}=\sum_{i=1}^{8}N_{i}u_{i}\\ v_{p}=\sum_{i=1}^{8}N_{i}v_{i}\\ w_{p}=\sum_{i=1}^{8}N_{i}w_{i} \end{array}\right.$$

where $N_{i}$ is the finite element shape function, satisfy:

(21) $$\begin{array}{c} N_{1}=\frac{1}{8}(1+\frac{x}{a})(1-\frac{y}{b})(1-\frac{z}{c}),N_{2}=\frac{1}{8}(1+\frac{x}{a})(1+\frac{y}{b})(1-\frac{z}{c})\\ N_{3}=\frac{1}{8}(1-\frac{x}{a})(1+\frac{y}{b})(1-\frac{z}{c}),N_{4}=\frac{1}{8}(1-\frac{x}{a})(1-\frac{y}{b})(1-\frac{z}{c})\\ N_{5}=\frac{1}{8}(1+\frac{x}{a})(1-\frac{y}{b})(1+\frac{z}{c}),N_{6}=\frac{1}{8}(1+\frac{x}{a})(1+\frac{y}{b})(1+\frac{z}{c})\\ N_{7}=\frac{1}{8}(1-\frac{x}{a})(1+\frac{y}{b})(1+\frac{z}{c}),N_{8}=\frac{1}{8}(1-\frac{x}{a})(1-\frac{y}{b})(1+\frac{z}{c}) \end{array}$$

As shown in Figure 5b, the displacements of each node at the boundary of the meso-scale model, i.e., the boundary condition of the meso-scale model, can be obtained from the displacements of the eight nodes of the macro-scale element combined with the finite element shape function of Equation (21). Based on displacement continuity theory and element interpolation theory for the transfer of boundary condition at macro–meso-scale, a cross-scale inheritance tool has been developed in this paper, which can quickly and accurately assign displacement field from macro-scale outputs to meso-scale model.

## 5. Surface Roughness Evolution of Drawing Process

A macro–meso-scale modelling framework is developed in this paper, which is a combination of a crystal plasticity finite element modelling, a fluid–solid interaction modelling, and a macro–meso-scale boundary condition. Then, this framework was numerically realized and applied to an aluminum alloy tube drawing process.

### 5.1. Macro-Scale Model of Aluminum Alloy Tube Drawing Process

**Figure 6:**
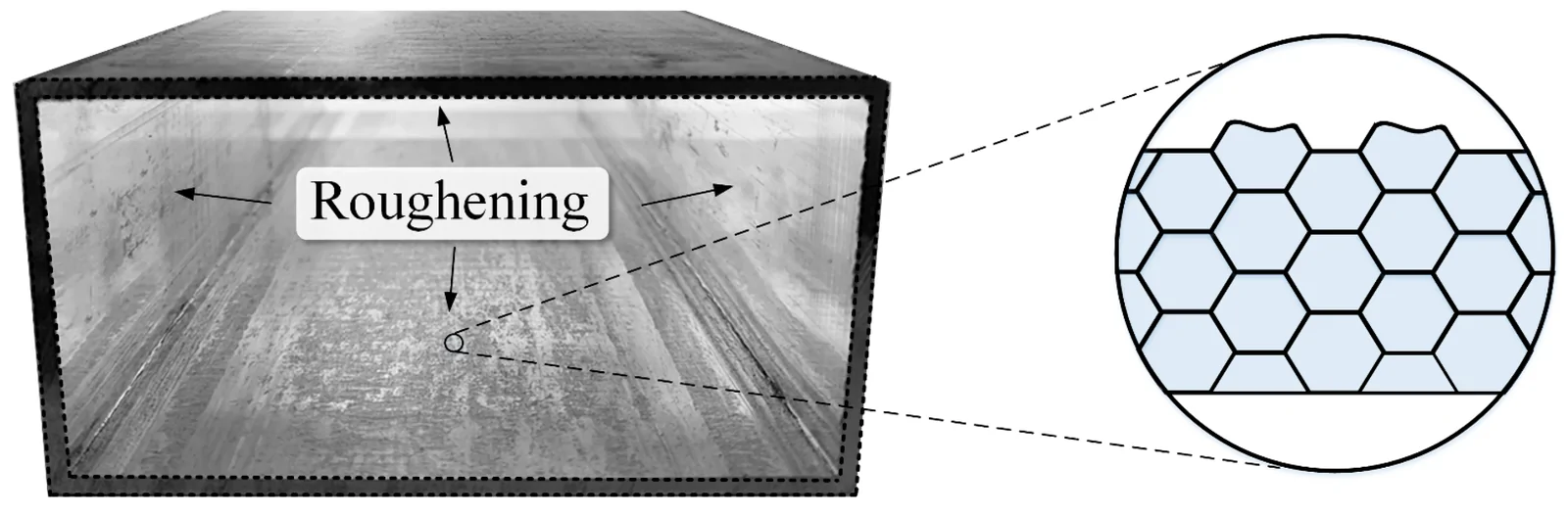
Roughening phenomena of the waveguide tube during the drawing process.

As the main medium for microwave transmission, waveguide tubes are widely used in aviation, aerospace, radar, telemetry and other fields [63,64]. As shown in Figure 6, the traditional forming method would result in severe roughening phenomena on the inner surface of the waveguide tube. The surface roughness of the waveguide tube has an important influence on the electromagnetic energy consumption and conductor loss during microwave transmission [1,65,66]. Due to the special characteristics of the environment in which waveguide tubes are used, not only do they require a high degree of dimensional accuracy, but they also place severe limit on the inner surface quality (surface roughness *Sq* ≤ 1.25 μm) [67]. The geometry of the waveguide tube is such that it is not suitable for mechanical machining of the internal surfaces. It is therefore necessary to obtain the inner surface of the waveguide with a high degree of accuracy by plastic machining technique.

**Figure 7:**
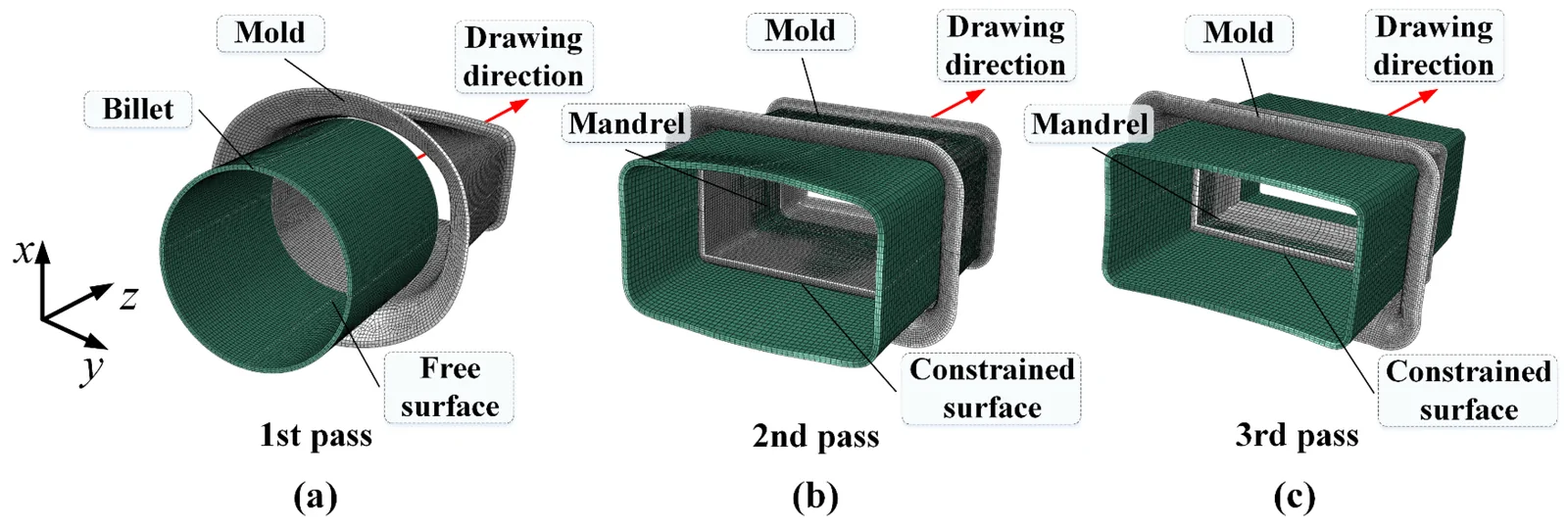
Drawing forming process for waveguide tube: (**a**) the first pass of forming, (**b**) the second pass of forming, (**c**) the third pass of forming.

As shown in Figure 7, the plastic forming process of the waveguide tube studied in this paper is divided into three stages, the first stage being empty drawing (no mandrel drawing), the second and third stages being drawing with mandrels. The surfaces of workpieces can be classified into two categories, free surface and constrained surface, according to their contact state during plastic deformation [37,68]. Therefore, the inner surface of the waveguide tube is a free surface in the empty drawing process, while the inner surface of the waveguide is a constrained surface during the drawing process with mandrels.

**Figure 8:**
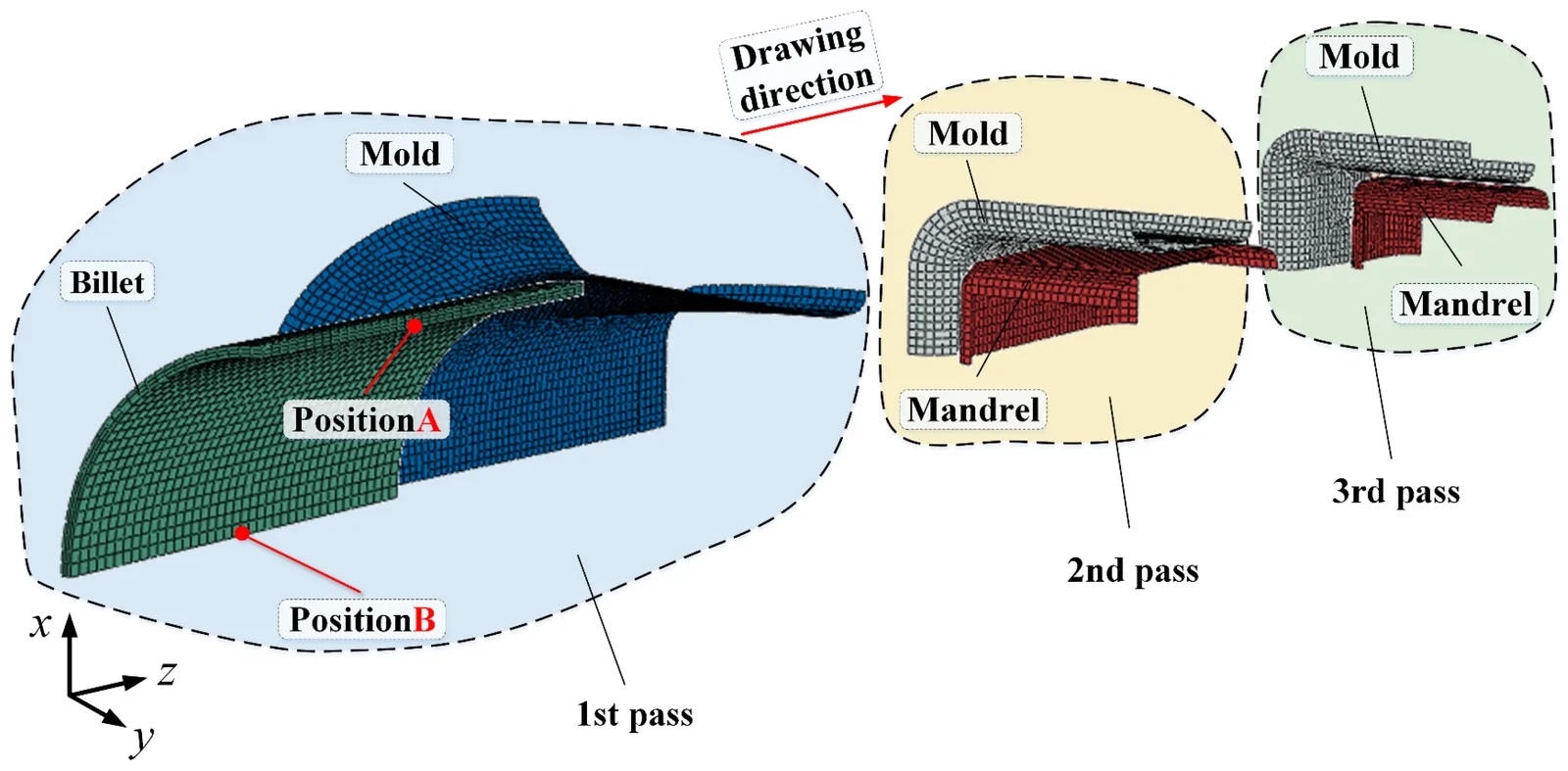
Macro-scale finite element model of rectangular waveguide tube drawing process.

As the waveguide tube, die and mandrel have a symmetrical structure, the macro-drawing finite element model can be taken as a quarter model for analysis with symmetry conditions constrained in the *x-o-z* and *y-o-z* planes. The macroscopic finite element model constructed for the three-pass drawing process of the rectangular waveguide tube is shown in Figure 8, where the *z*-direction is the drawing direction. The positions where severe roughening occurs are on the inner surface at symmetrical points, i.e., positions A and B of the quarter model shown in Figure 8. Therefore, a coupled macro–meso model will be developed for these two typical positions, focusing on their surface roughness evolution during the three-pass drawing process.

### 5.2. Macro–Meso-Scale Model of Surface Roughness Evolution

**Figure 9:**
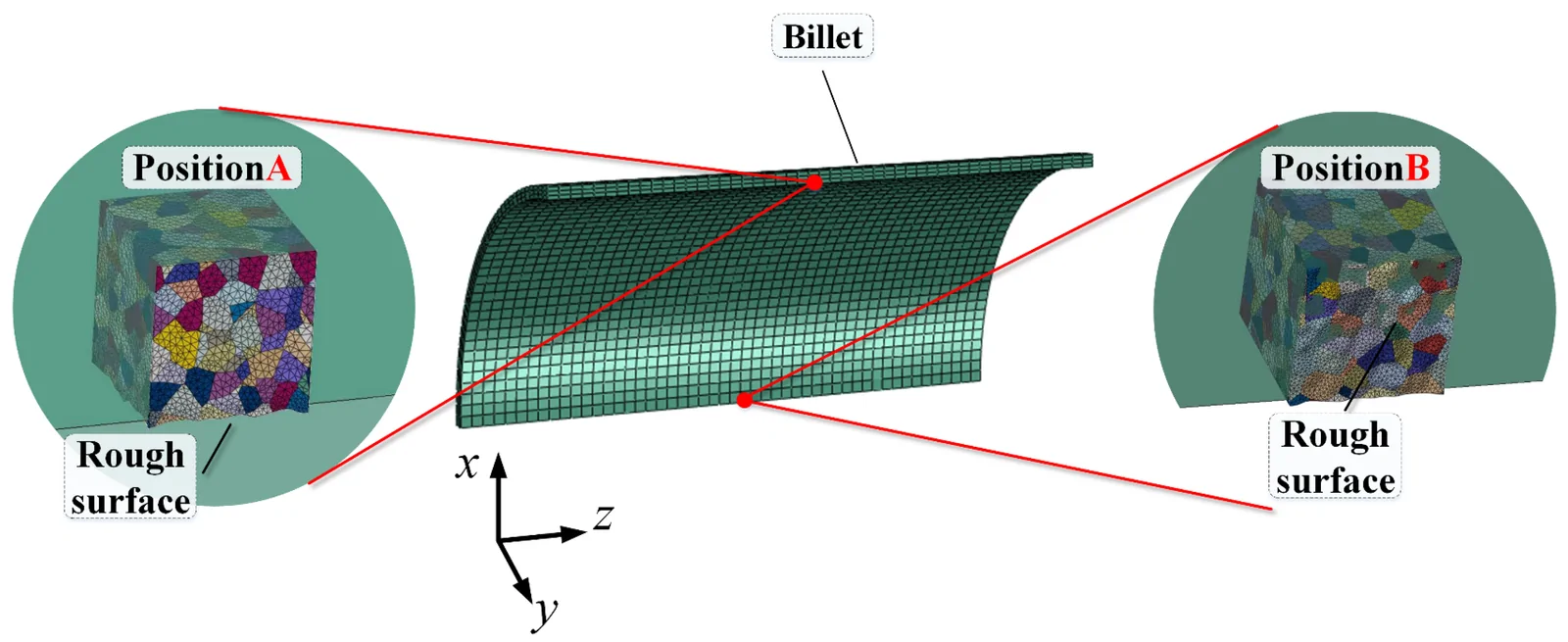
Macro and meso coupling model of rectangular waveguide tube drawing process.

In the previous section, a macroscopic model of the waveguide forming process was created which provided field information of the material flow. In this section, the meso-scale detailed models were created for key positions A and B, and the real measured surface topographies of positions A and B were assigned to the surfaces of the meso-scale models, as shown in Figure 9. These two scale models transfer information via the developed cross-scale inheritance tool, thus enabling coupled macro–meso modelling of the surface topography evolution for the waveguide tube drawing process.

### 5.3. Simulation Results of Drawing Process

**Figure 10:**
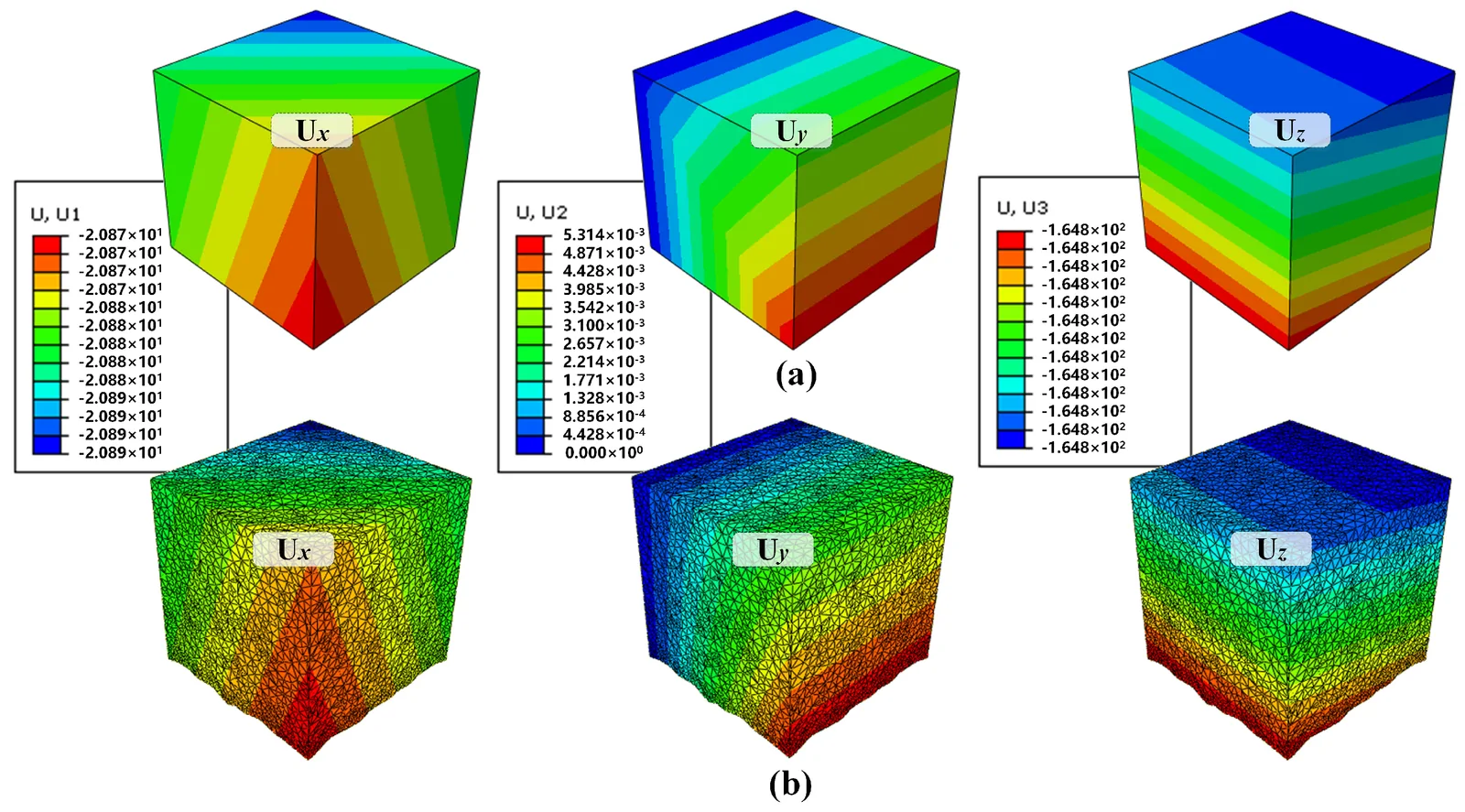
Comparison of the displacement fields between (**a**) the macroscopic model and its corresponding (**b**) mesoscopic model at position A.

The displacement results of the macro-scale model and the meso-scale model at typical position A are shown in Figure 10, where it can be found that the displacement results of the meso-scale model after the coupled macro–meso analysis are in good agreement with those of the corresponding macroscopic model, with a maximum displacement error of 1.0428 × 10^−4^ mm. Therefore, the meso-scale model provides an accurate representation of the deformation process.

The surface topography after the first pass deformation is coupled with the FSI model to take into account the effect of lubrication when simulating the roughness evolution of the constrained surface for the second and third pass drawing process. The material parameters for lubricants used in this paper are summarized in Table 2.

**Figure 11:**
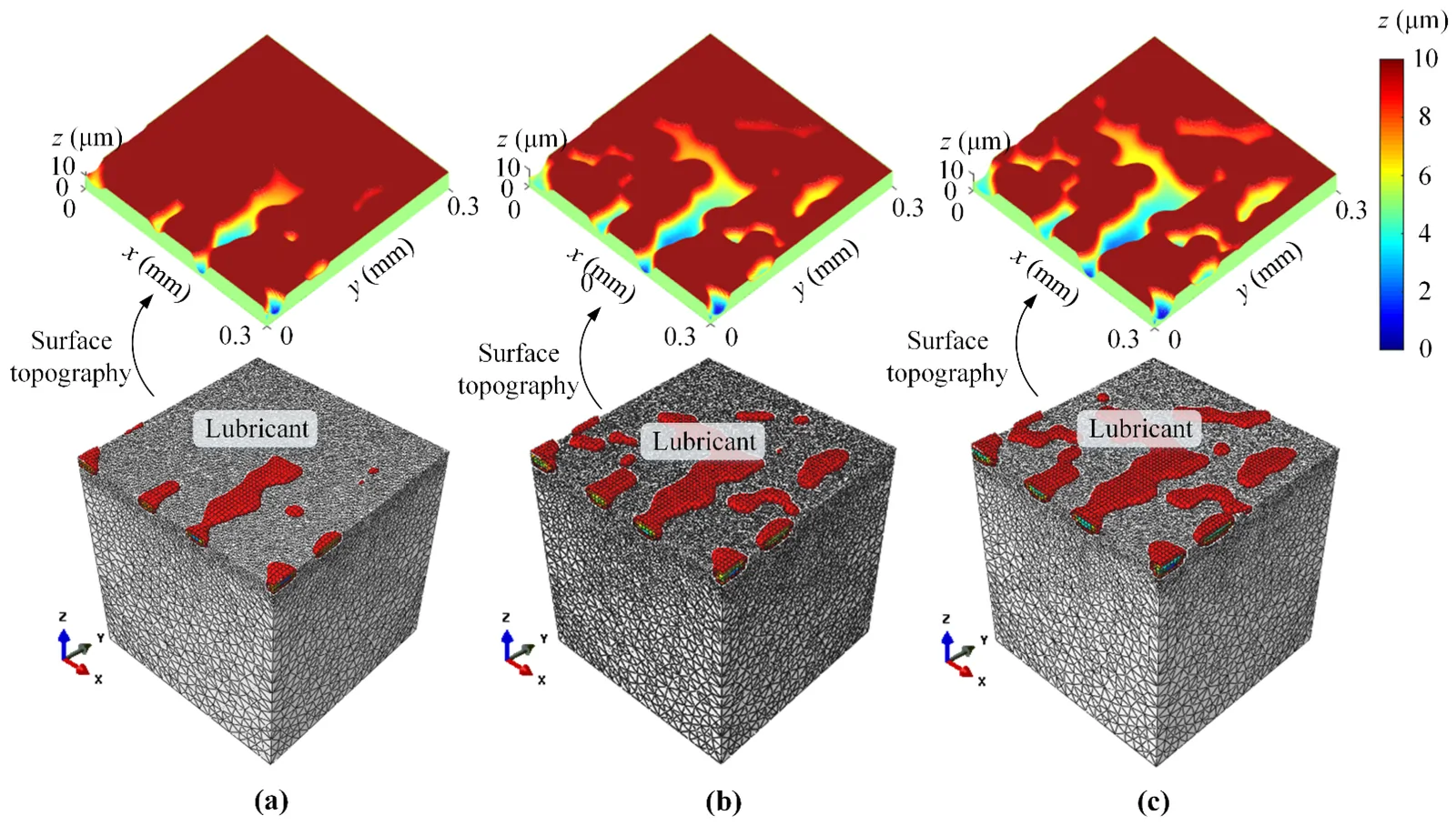
Simulated surface topography of the waveguide at position A after drawing using different lubrications: (**a**) water-based lubricant, (**b**) YOK 3000 lubricant, (**c**) No. 38 cylinder oil.

The simulation results of the internal surface topography of the waveguide tube at typical position A after three pass drawing with different lubricants are shown in Figure 11, where it can be seen that the closed cavities on the surface increase as the viscosity of the lubrication increases. Our research on constrained surfaces has found that [37] as deformation proceeds, the surface peaks on the workpiece surface that are involved in deformation gradually become more numerous and the undeformed surface valleys gradually form closed cavities. Then, as plastic deformation continues, the closed cavities are gradually flattened, while the lubricating medium in the closed cavities plays an important role in impeding the flattening behaviour of the surface.

**Figure 12:**
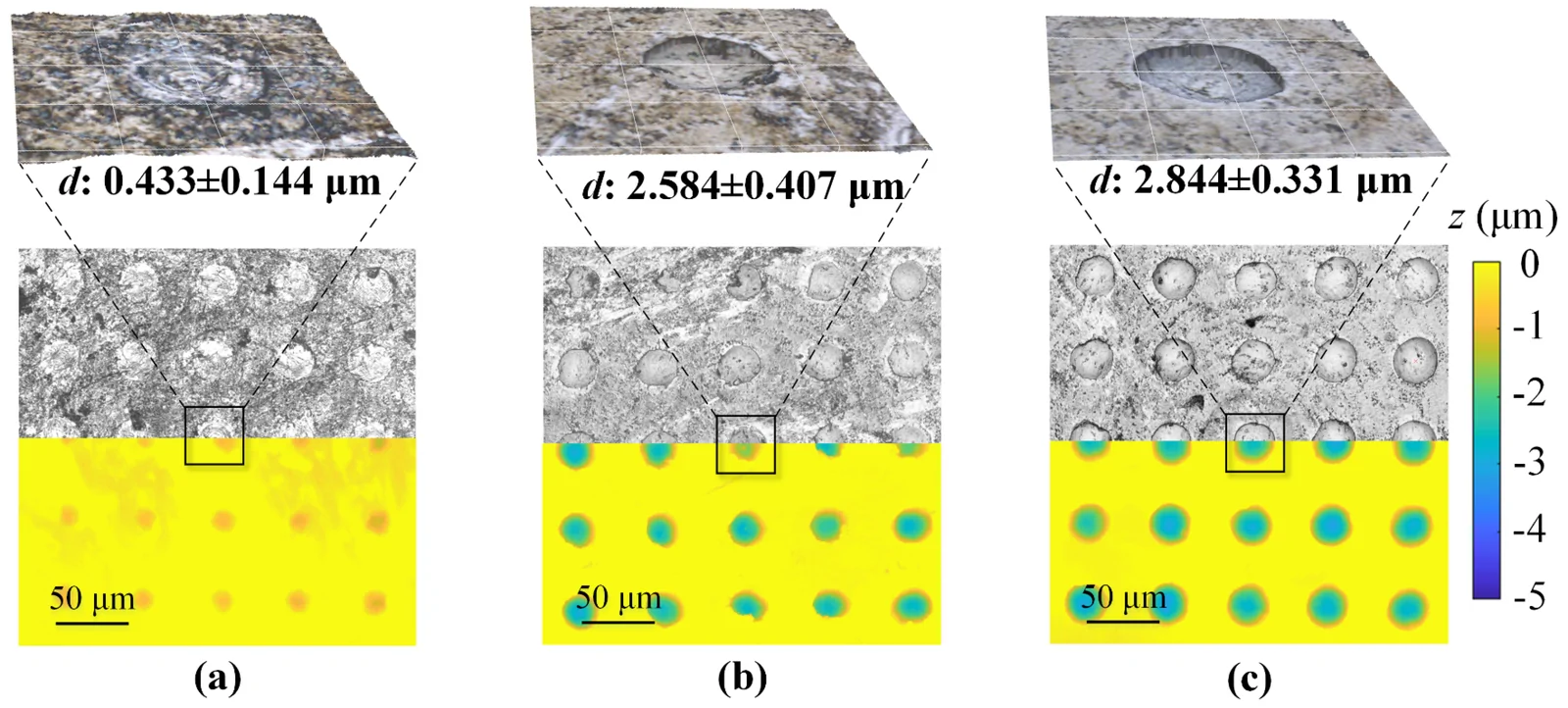
Deformation results of closed cavities under different lubrication conditions: (**a**) dry friction, (**b**) water-based lubrication, (**c**) oil-based lubrication.

For dry friction, a Coulomb friction coefficient of 0.15 (typical for aluminum–steel contact [69]) was used. For lubricated conditions, the fluid–solid interaction model inherently accounts for friction through the lubricant’s viscosity and hydrodynamic pressure. Under dry friction conditions, the deformation behaviour is presented in Figure 12a. As the die indentation progresses, the deepest point of the cavity shifts upward toward the contact interface, causing the cavity depth to diminish continuously until it eventually reaches zero. The introduction of a lubricating medium markedly suppresses this cavity-flattening effect: the average cavity depth under dry friction measures 0.433 μm, whereas under water-based lubrication and oil-based lubrication, as depicted in Figure 12b and Figure 12c, it is 2.584 μm and 2.844 μm, respectively.

In this paper, the contour maps of surface roughness are constructed based on a predictive model of back propagation neural network (BPNN) in order to investigate the effect of intrinsic factors (grain size, grain orientation and initial surface roughness) and extrinsic factors (deformation path, strain accumulation and lubrication conditions) on the evolution of surface roughness. The BPNN architecture is composed of an input layer with three neurons (corresponding to the three input factors), a single hidden layer with ten neurons and a sigmoid activation function, and an output layer with one neuron and a linear activation function to predict the surface roughness parameter *Sq*. The dataset was randomly partitioned into training (70%) and validation (30%) subsets. The network was trained using the Levenberg–Marquardt algorithm with mean squared error (MSE) as the loss function. Training was terminated when the MSE fell below 10^−5^ or the maximum epoch count of 1000 was reached. Based on these contour maps, a series of reasonable optimizations can be developed to achieve the high requirements for the surface roughness the waveguide tube.

**Figure 13:**
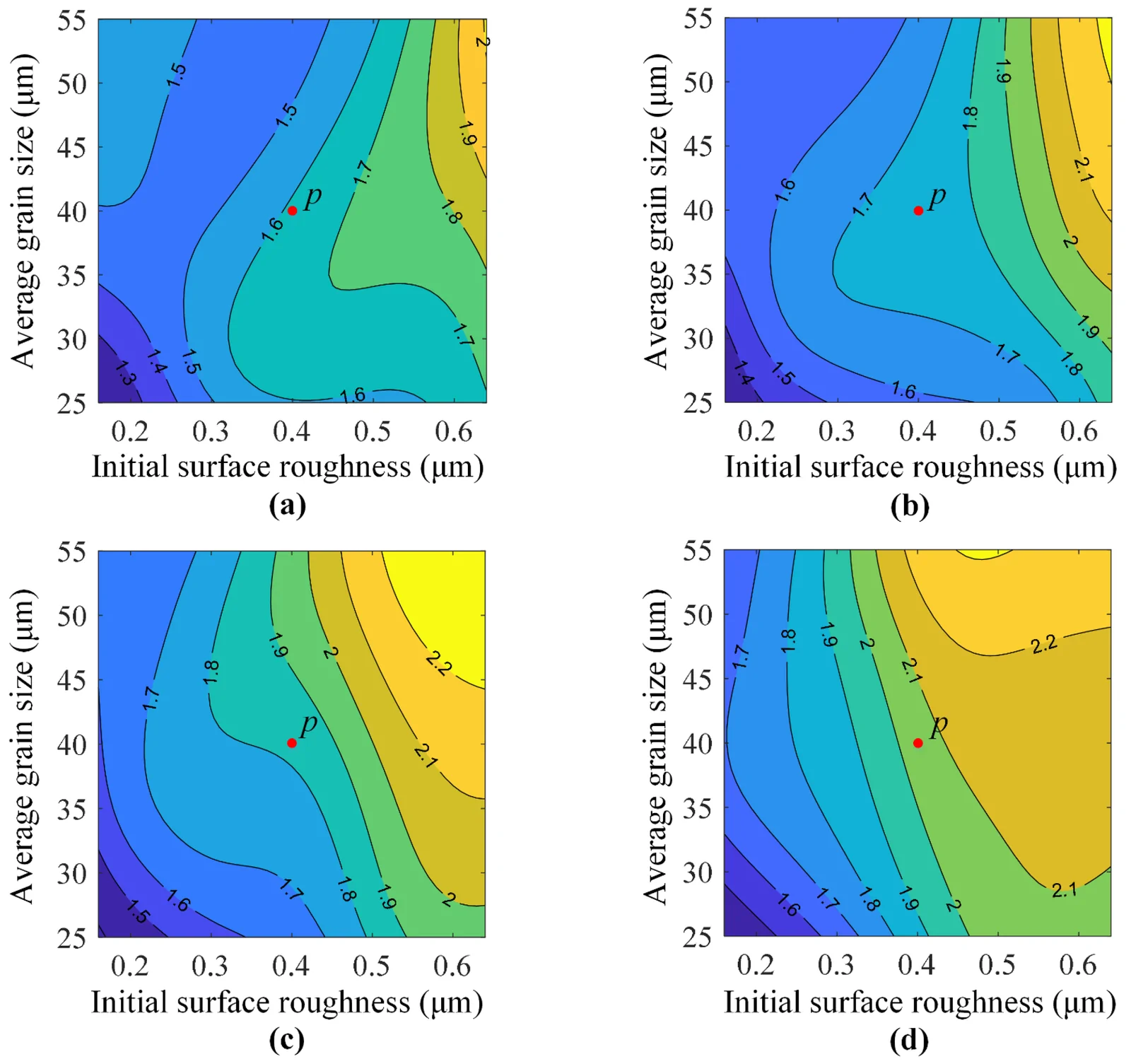
The contour maps of intrinsic factors: (**a**) 20% grain random orientation, (**b**) 40% grain random orientation, (**c**) 60% grain random orientation, (**d**) 80% grain random orientation.

The contour maps between the surface roughness of the simulated waveguide tube and intrinsic factors (grain size, grain orientation and initial surface roughness) are shown in Figure 13. It can be found that when the metal has different random grain orientations, the contour maps of surface roughness are always lowest in their lower left region, conversely, highest in its upper right region. This suggests that rectangular waveguide tubes with low surface roughness can be obtained after three-pass drawing process by adjusting the preparation of the tube billets to have a low initial surface roughness and a fine grain size. As shown in Figure 13, the roughness evolution at the same position p in the four contour maps was also analyzed, and it was found that the surface roughness at position p gradually increased as the random grain orientation increased. Therefore, by controlling the material properties of the tube billets to have a lower random grain orientation, waveguide tubes with a lower surface roughness can also be obtained.

**Figure 14:**
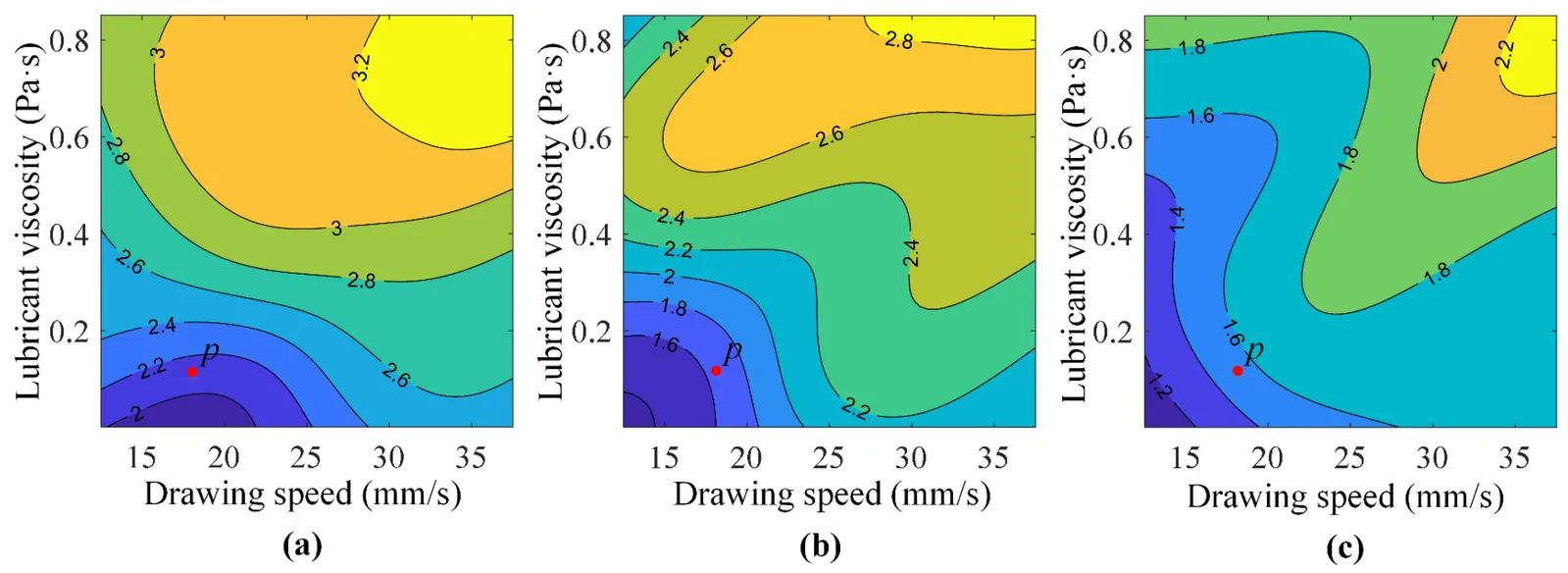
The contour maps of extrinsic factors: (**a**) 2-pass forming, (**b**) 3-pass forming, (**c**) 4-pass forming.

Next, the influence of extrinsic factors, i.e., deformation path, strain rate and lubrication condition, on the evolution of surface roughness is discussed in this paper. These external factors can be expressed in terms of the process factors, such as drawing passes, drawing speed and lubricant viscosity. The contour maps between the surface roughness of the simulated waveguide tube and process factors (drawing passes, drawing speed and lubricant viscosity) are shown in Figure 14. It can also be found that when the drawing process is designed for different passes, the contour maps of surface roughness are always lowest in their lower left region, conversely, highest in its upper right region. This suggests that by controlling the drawing process factors to have a lower drawing speed and a lower lubricant viscosity, rectangular waveguide tubes with low surface roughness can be achieved. As shown in Figure 14, the roughness evolution at the same position p in the three contour maps was also analyzed, and it was found that the surface roughness at position p gradually decreased as the number of drawing passes increased. Therefore, by adjusting the drawing process to have more forming passes, it is also possible to reduce the surface roughness of rectangular waveguide tubes. It was noted that the reduction in roughness was 29.1% when changing from two to three forming passes and 7.9% when increasing from three to four forming passes. Therefore, when designing the number of forming passes for the drawing process, it is important not to blindly pursue more forming passes, but to balance the economics of die manufacture with the improvement of surface roughness.

In summary, the roughness evolution of rectangular waveguide tubes is simulated at the macro–meso-scale. And contour maps of the influence of internal factors and external factors on the roughness evolution are constructed by BPNN method. From a practical point of view, the grain size and the initial surface roughness of tube billet, the drawing speed and the lubricant of forming process are optimized in the next experiments due to the consideration of the economy and time of modifying the various factors.

### 5.4. Experiment Results of Drawing Process

At present, with the rapid development of computer technology, the application of finite element techniques for the numerical simulation and analysis of plastic forming processes has made great progress and has become the mainstream method of research in the field of plastic forming. However, the reliability and accuracy of the numerical simulation still depend on the reasonable establishment of the physical model, initial conditions and boundary conditions. Plastic forming is actually a very complex process, which includes physical and geometrical non-linearities as well as complex loading histories and boundary conditions, so it is difficult to obtain a very accurate result from finite element calculations under such conditions. Therefore, experimental methods are still a valid and necessary method. Not only can it be used to check the correctness of the numerical calculations, but the experimental study itself is also an effective way to quantify the stress–strain state of plastic forming and the surface roughness of the workpiece. In this paper, an experimental study of a drawing process for rectangular waveguide tube in 6061 aluminum alloy will be carried out to verify the effectiveness of the provided optimization scheme.

**Figure 15:**
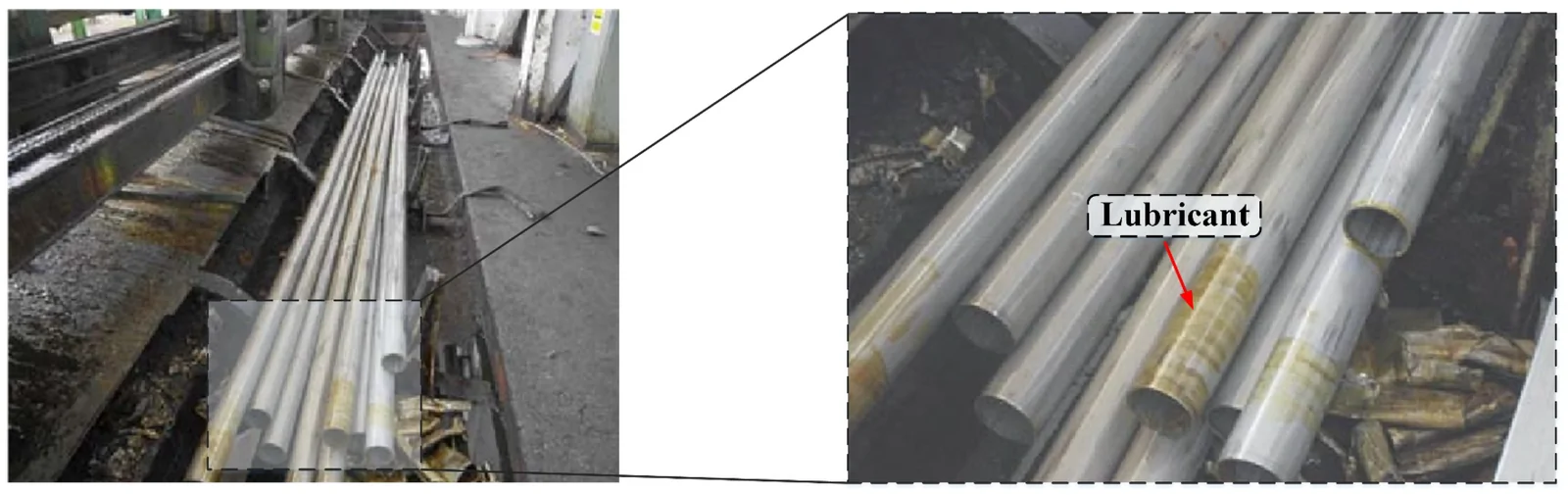
Round tube billets for rectangular waveguide tube production tests.

The drawing billet is a round tube with a diameter of 92.8 mm, a wall thickness of 2.15 mm and a length of 7 m, as shown in Figure 15. The preparation process is as follows: ingot casting → hot extrusion → annealing treatment → cold rolled round tube → pickling treatment → round tube billet. Before the drawing of the rectangular waveguide tube begins, the billet is first soaked in a lubricating medium of No. 38 cylinder oil for 24 h. It is then suspended to remove the excess lubricating medium. Finally, a uniformly distributed lubricant film is formed on the surface of the billet to enhance the drawing lubrication effect.

**Figure 16:**
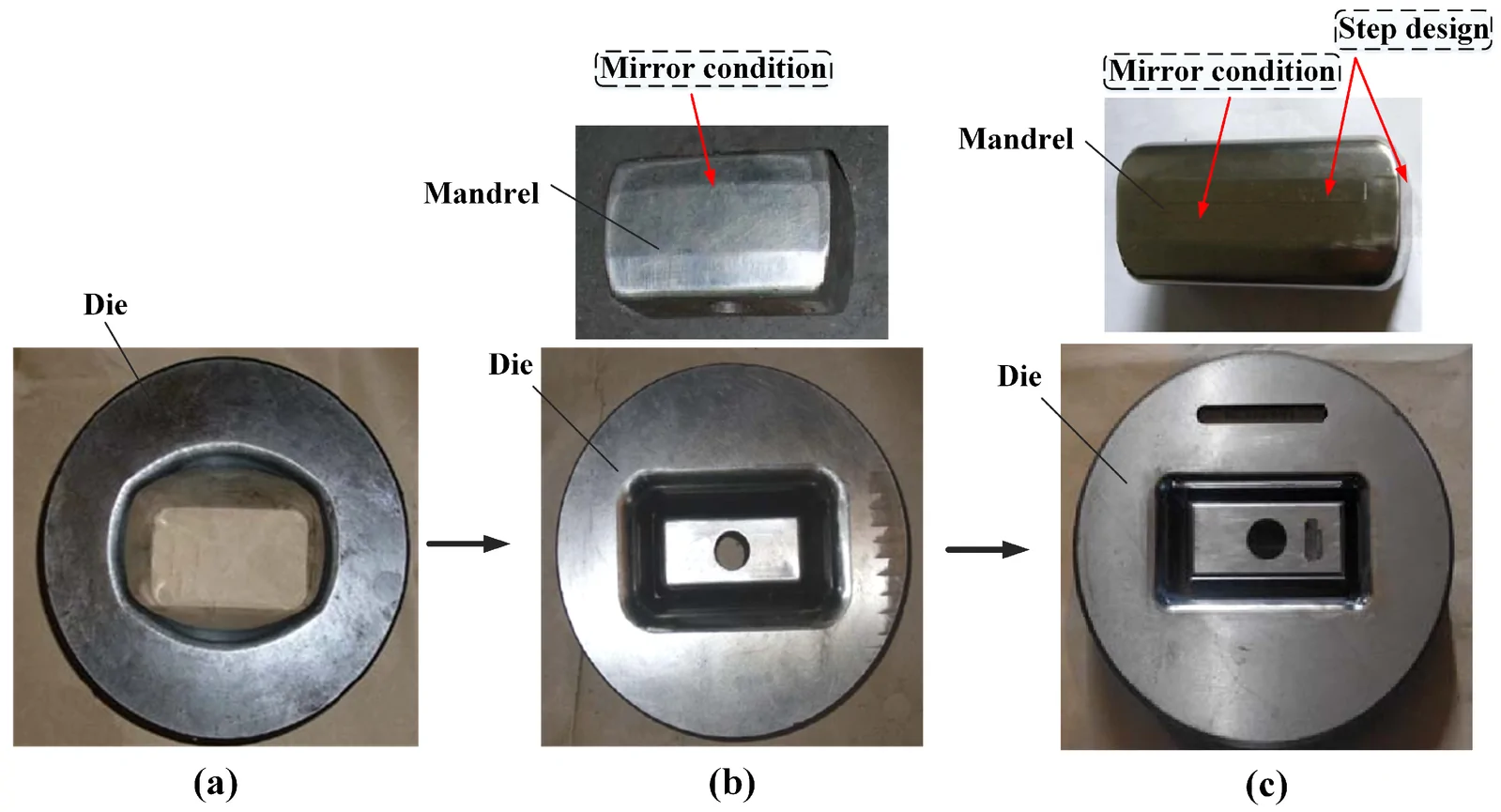
Dies and mandrels for rectangular waveguide tube drawing process. (**a**) 1-pass forming, (**b**) 2-pass forming, (**c**) 3-pass forming.

The die and mandrel for the rectangular tube drawing process are shown in Figure 16. The material of the die and mandrel is Cr12MoV cold work die steel with a heat treatment hardness of 59~63 HRC. The mandrels are polished to a surface accuracy of *Ra* 0.2 μm to achieve a basic “mirror” condition, while an innovative step design is added to guarantee good internal surface quality of the finished tube.

**Figure 17:**
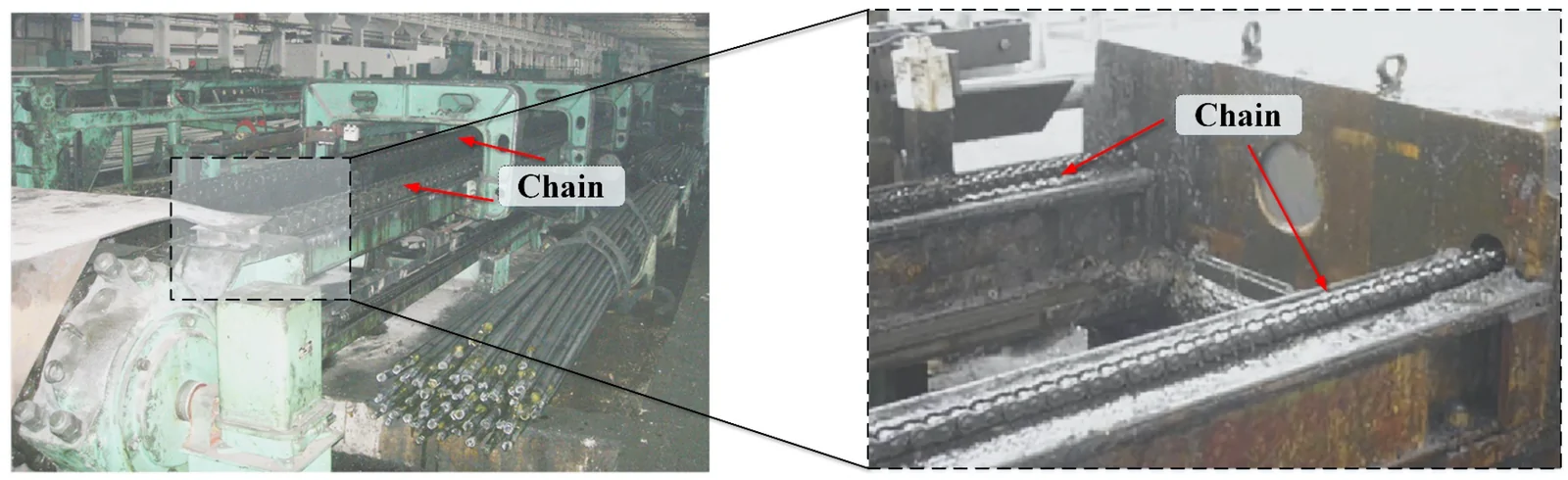
Chain-puller equipment for rectangular waveguide tube drawing tests.

The most widely used drawing equipment is the chain puller, as shown in Figure 17. This equipment is simple in structure, easy to operate and highly adaptable, enabling the forming of tubes, bars and profiles. For the drawing tests with this equipment, a slow pulling speed of 12.5 mm/s was used.

**Figure 18:**
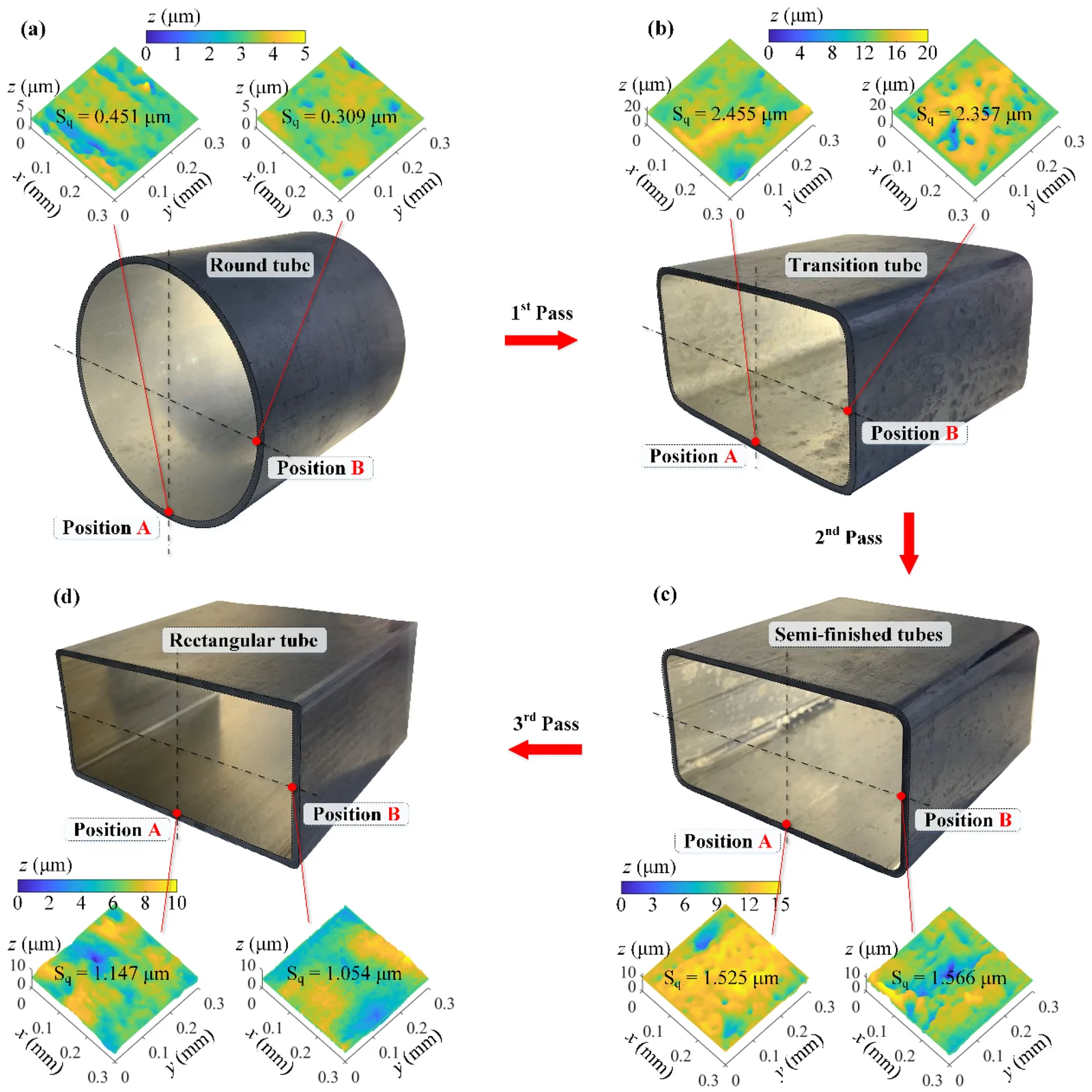
Waveguide shape and measured roughness obtained in each pass: (**a**) round tube billet, (**b**) transitional rectangular tube, (**c**) semi-finished rectangular tube, (**d**) finished rectangular tube.

The waveguide shape from each drawing pass is shown in Figure 18. The shape of the rectangular waveguide tube after three-passes forming are acceptable and the width, height and wall thickness of the finished rectangular tube are within the tolerances required by the design. And as shown in Figure 18d, it can be clearly seen that the severe roughening of the inner surface of the finished rectangular tube has been greatly improved. The real surface topography was measured using an Olympus OLS4000 confocal laser scanning microscope (CLSM) (Olympus Corporation, Tokyo, Japan) in accordance with the ISO 25178 [8]. To ensure repeatability, five measurements were performed at different locations on the sample; the average value was taken as the final result. The relative deviation among the repeated measurements was consistently below 5%, confirming high measurement reproducibility.

The surface roughness of the tube at typical positions A and B for each pass. The average roughness *Sq* of the round tube at key positions A and B was 0.380 μm, as shown in Figure 18a. The round tube is formed through the first pass of without-mandrel drawing to become a transition rectangular tube with an average roughness *Sq* of 2.406 μm at positions A and B, as shown in Figure 18b. Then, the transitional rectangular tube is formed with mandrel drawing in the second pass to become a semi-finished rectangular tube with an average roughness *Sq* of 1.545 μm at typical locations A and B, as shown in Figure 18c. The finished rectangular tube is formed by the third drawing pass, and the measured average roughness *Sq* at typical positions A and B was 1.100 μm, as shown in Figure 18d. From the aspect of surface roughness, the surface quality of the rectangular waveguide tube after forming with the optimized solution meets the design requirements.

## 6. Conclusions

In this paper, a macro–meso-scale modelling framework is developed, which couples crystal plasticity finite element modelling, fluid–solid interaction modelling, and a macro–meso-scale boundary condition. This framework was numerically realized and applied to an aluminum alloy tube drawing process, and the effects of intrinsic factors (grain size, grain orientation and initial surface roughness) and extrinsic factors (deformation path, strain rate and lubrication condition) on surface roughness evolution are investigated. The results show that the surface roughness of tube drawing process is controlled by several factors: grain size, grain orientation, initial surface roughness, deformation path, strain rate and lubrication condition. From a practical point of view, these are effective strategies to improve surface quality by reducing grain size, lowering initial surface roughness, decreasing the strain rate and using low-viscosity lubricants. The main conclusions are as follows:

1. A method of constructing a surface topography using the real rough surface of the workpiece as a finite element model is presented. Detailed information of the real surface topography, including the number and exact locations of peaks and valleys on the surface, are accurately captured in the constructed real surface model.
2. The displacement results of the meso-scale model after the coupled macro–meso analysis are in good agreement with those of the corresponding macroscopic model, with a maximum displacement error of 1.0428 × 10^−4^ mm. Therefore, the meso-scale model provides an accurate representation of the deformation process.
3. Rectangular waveguide tubes with low surface roughness can be obtained after three-pass drawing process by adjusting the preparation of the tube billets to have a low initial surface roughness, a fine grain size and a lower random grain orientation.
4. By controlling the drawing process factors to have lower drawing speed and lower lubricant viscosity, rectangular waveguide tubes with low surface roughness can be achieved. The reduction in roughness was 29.1% when changing from two to three forming passes and 7.9% when increasing from three to four forming passes.
5. The optimized drawing process produced rectangular waveguide tubes with acceptable geometry and surface quality.
6. The modelling methodology and the numerical skill proposed in the paper could be extended to further research on the relationships between surface roughening and localized necking or shear banding.

## Figures and Tables

**Table 1:** Material parameters for crystal plasticity constitutive model of 6061 aluminum alloy [37].

Material Parameters	Elastic Parameters			Plastic Parameters					
	$C_{11}$ (GPa)	$C_{12}$ (GPa)	$C_{44}$ (GPa)	m	${\dot{\gamma }}_{0}$	$h_{0}$ (MPa)	$\tau_{0}$ (MPa)	$\tau_{s}$ (MPa)	q
value	108.2	61.3	28.5	0.05	1	96	23.5	54	1.1

**Table 2:** Material parameters of the lubricants.

Lubricant	Density	Dynamic Viscosity	Sound Velocity	Mie–Grüneisen Ratio	Slope Parameter
	ρ (kg/m^3^)	μ (Pa·s)	$c_{0}$ (m/s)	Γ	s
Water-based lubricant	1000	0.001	1450	0	0
YOK 3000 lubricant oil	880	0.0598	1401	0	0
No. 38 cylinder oil	880	0.8556	1401	0	0

## Data Availability

The original contributions presented in this study are included in the article. Further inquiries can be directed to the corresponding author.
